# Global burden and cross-country inequalities of gallbladder and biliary tract cancer in adults aged 45 years and older from 1990 to 2021: population-based study

**DOI:** 10.3389/fonc.2025.1676636

**Published:** 2025-10-09

**Authors:** Simeng Lei, Yangkai Fu, Bo Zhang, Hanwen Yang, Yang Hu, Zhili Ji

**Affiliations:** Department of Hepatobiliary and Pancreaticosplenic Surgery, Beijing Chaoyang Hospital, Capital Medical University, Beijing, China

**Keywords:** gallbladder and biliary tract cancers, global burden of disease, risk factors, decomposition analyses, frontier analysis

## Abstract

**Objective:**

Gallbladder and biliary tract cancers (GBTC) remain a global health burden, particularly in adults aged 45 years and older. Understanding trends, regional disparities, and risk factors is crucial for guiding public health strategies. This study aimed to evaluate the global, regional, and national patterns of GBTC burden from 1990 to 2021, with a focus on socio-demographic variations and cross-country inequalities.

**Methods:**

Data were obtained from the Global Burden of Disease (GBD) 2021 study, covering 204 countries and territories. Age-standardized incidence rates (ASIR), age-standardized prevalence rates (ASPR), age-standardized mortality rates (ASMR), and age-standardized disability-adjusted life years rates (ASDR) were calculated. Temporal trends were assessed using estimated annual percentage changes (EAPC). Frontier and decomposition analyses were conducted to identify drivers of changes, and the role of high body mass index (BMI) and population aging was examined.

**Results:**

In 2021, the global ASIR of GBTC was 9.04 per 100,000, showing an annual decline of 0.453%. However, ASPR increased slightly by 0.240% per year. The ASDR and ASMR declined annually by 1.080% and 0.903%, respectively. High-SDI regions exhibited increasing incidence due to epidemiological transitions, while low-SDI regions were more affected by aging populations. Frontier analysis revealed widening disparities between high- and low-SDI countries. High BMI was identified as a major risk factor, contributing significantly to DALYs and mortality, particularly in high-income regions such as North America. Individuals aged 50–54 had the highest burden attributable to high BMI, accounting for 13.08% of DALYs and 13.06% of deaths. Cross-country analysis indicated persistent inequalities, with higher incidence concentrated in high-SDI countries. Although relative inequalities in prevalence and mortality narrowed modestly between 1990 and 2021, substantial disparities across SDI levels remain.

**Conclusion:**

Although the global burden of GBTC has declined in mortality and DALYs, incidence trends remain concerning, especially in high-SDI countries. Regional disparities persist and have widened over time, with high BMI and population aging as key drivers. While certain indicators suggest modest narrowing of inequalities, large gaps between SDI regions remain. Public health policies should focus on mitigating these disparities, particularly by addressing high BMI and strengthening interventions in low-SDI regions to further reduce the burden of GBTC.

## Introduction

Gallbladder and biliary tract cancers (GBTC) comprises a group of highly heterogeneous malignant tumors originating from the epithelial cells of the biliary tract (1). GBTC is characterized by high malignancy, frequent recurrence and metastasis, and poor prognosis. A significant proportion of patients are diagnosed at an advanced stage, with only a small subset eligible for surgical intervention. The 5-year survival rate for these patients has been reported to range from 5% to 15% (2, 3).

Despite its relatively low overall prevalence, GBTC continues to impose a substantial health burden on specific high-risk populations (4). Numerous studies have explored various risk factors associated with GBTC, including cholelithiasis, chronic biliary inflammation, high body mass index, metabolic syndrome, and specific infectious agents, such as liver fluke and hepatitis B virus (5, 6). However, many studies have primarily focused on analyzing case data from localized areas, and the conclusions drawn are often controversial. Furthermore, epidemiological risk factors vary between ethnic groups, potentially reflecting molecular differences among populations of patients with biliary malignancies. Metabolic syndrome, hepatitis C and alcoholism have been identified as significant epidemiological risk factors in the European American population with biliary malignancies (7). Conversely, higher epidemiological risk factors such as hepatitis B and biliary stones were observed in the Asia-Pacific population with biliary malignancies (8). A macro-level assessment from a global perspective, utilizing large sample data, is therefore recommended.

In recent years, molecularly targeted therapies and immune checkpoint channels have begun to significantly reshape the therapeutic landscape (9). However, the temporal trends of GBTC prevalence and disease burden at both global and regional levels have yet been systematically assessed, despite advancements in treatment modalities. Given the high prevalence of GBTC, there is a pressing need to examine the trends in prevalence among middle-aged and older adults (10). Currently, there is a scarcity of studies addressing trends in the global burden of disease among individuals aged 45 years and older.

This study aims to analyzes the global trends on GBTC among adults aged 45 years and older from 1990 to 2021. Using the most recent data from the Global Burden of Disease (GBD) 2021 study, exploring variations in disease burden across regions and countries with different levels of socioeconomic development (6, 11). By highlighting these trends and their associations with global health disparities and aims to inform evidence-based public health policies and promote the efficient allocation of healthcare resources worldwide.

## Methods

### Data acquisition

This study utilizes data from the GBD 2021 study, a comprehensive resource that assesses the global and regional impact of 371 diseases, injuries, and impairments, and 88 risk factors, across 204 countries and territories from 1990 to 2021 (12). Compiled with contributions from thousands of international collaborators, the GBD 2021 dataset offers detailed insights into the incidence, prevalence, mortality, and disability-adjusted life years (DALYs) associated with a wide range of health conditions. For this analysis, data specific to GBTC were extracted using the Global Health Data Exchange (GHDx) platform (https://vizhub.healthdata.org/gbd-results/). This dataset includes information disaggregated by age, sex, geographic location, and risk-attributed DALYs and deaths. To explore the influence of economic and social factors on the burden of GBTC, additional data were retrieved from the Socio-Demographic Index (SDI) (https://ghdx.healthdata.org/gbd-2021). The SDI is a composite measure, annually updated by the GBD collaborative using current data on income per capita, educational attainment in the population aged 15 years and older, and fertility rate under age 25, and classifies countries into five development categories—low, low-middle, middle, high-middle, and high—ensuring temporal comparability from 1990 to 2021.

### Data sources and disease definition

The assessment of the global burden of GBTC was conducted using data from vital registration systems, hospital discharge records, claims data, and population-based studies. The disease was defined using codes C23, C24–C24.9in the 10th revision of the International Classification of Diseases and Injuries (ICD-10), and 156–156.9 in ICD-9. Severity classifications included asymptomatic, mild, moderate, and severe categories. Mortality estimates were derived using the Cause of Death Ensemble Model (CODEm) and were adjusted using CoDCorrect algorithm to ensure consistency across all causes of death. For non-fatal estimates (incidence, prevalence) were modeled using DisMod-MR 2.1, a Bayesian meta-regression model that ensures internal consistency across disease parameters. DisMod-MR 2.1 incorporated prior distributions and used a compartmental modeling framework to estimate incidence, prevalence, remission, and mortality simultaneously. Adjustments for study bias and non-standard case definitions were made using MR-BRT (Meta-Regression—Bayesian, Regularized, Trimmed), which enabled cross walking of heterogeneous data sources. Covariates included body mass index (BMI), alcohol use, dietary intake, healthcare access, and other region-specific social determinants to refine model estimates. Remission rates were estimated using healthcare access metrics and age as covariates. To ensure comparability across geographies and time, validation was performed through out-of-sample testing and comparison with empirical data, and estimates were smoothed using spatiotemporal Gaussian process regression (ST-GPR) in areas with sparse data. Ultrasonography-based prevalence data served as a reference standard for bias correction in non-imaging-based studies. This comprehensive, standardized approach allowed for reliable estimation of GBTC burden across demographic and socioeconomic spectra. Further details on the modeling can be found in the GBD 2021 methods appendices (https://www.healthdata.org/gbd/methods-appendices-2021).

### Estimation of disease burden

In 2021, a comprehensive analysis was conducted to assess the burden of GBTC using four key metrics: age-standardized incidence rates (ASIR), age-standardized prevalence rates (ASPR), age-standardized DALY rates (ASDR), and age-standardized mortality rates (ASMR). This evaluation also explored how demographic and socioeconomic factors, including age, sex, and the SDI, influenced the global and regional distribution of the disease burden. To examine long-term trends, the estimated annual percentage change (EAPC) for these rates was assessed during the study period. The EAPC serves as a standardized tool to track changes in disease burden over time. It is calculated using a log-linear regression model applied to the natural logarithms of age-standardized rates (ASRs) with the equation: ln(ASR)=a + bx + e. Here, ln(ASR) represents the natural logarithm of the ASR, x corresponds to the calendar year, a is the intercept, b is the slope, and e is the error term. The EAPC is derived as: EAPC=(exp (b) - 1) × 100, with 95% confidence intervals (CI) derived from the regression (13). A trend is considered decreasing if the upper CI limit is below 0 and as increasing if the lower CI limit exceeds 0. This analytical framework provides a detailed understanding of the GBTC burdens through incidence, prevalence, mortality, and DALYs over 32 years. It highlights disparities across different age groups and socioeconomic levels, offering valuable insights for developing targeted public health interventions to address the global and regional challenges of this disease. Additionally, in the GBD 2021 study, the impact of high body-mass index on DALYs and deaths of GBTC was analyzed, and the proportion of the burden attributable to this risk factor was reported.

### Frontier analysis

To evaluate the relationship between the burden of GBTC and the level of socio-economic development, represented by the SDI, we employed a frontier analysis to develop a model based on age-standardized rates (ASR). Unlike traditional regression models, frontier analysis captures the non-linear relationship between SDI and disease burden while identifying multidimensional drivers. In simpler terms, frontier analysis helps us understand how socio-economic factors influence cancer rates by identifying the “best possible” rates of cancer that a country could achieve given its level of development. This approach establishes the theoretical minimum ASR achievable by each country or region at its current level of development, setting a benchmark for optimal performance. Think of it as finding the best possible cancer rates for a country given what it has to work with, like its healthcare system or economic resources It also quantifies the gap between actual burden and the theoretical minimum, thereby highlighting areas for improvement (14). In other words, we measure how far each country is from reaching its optimal cancer rate, helping to identify areas that need more attention. We combined locally weighted regression with local polynomial regression, applying a smoothing span of 0.3 to generate a smooth, non-linear boundary curve between SDI and ASR. To ensure robustness, 1,000 bootstrap samples were conducted, and the mean ASR for each SDI value was calculated. Finally, by measuring the absolute distance between the actual ASR of each country in 2021 and the boundary curve, we assessed the potential for reducing the gallbladder and biliary tract cancer burden across different countries. This last step involves comparing how each country’s cancer rate compares to the optimal curve we created, helping us understand the potential for improvement.

### Decomposition analysis

To identify the factors driving changes in GBTC cases, a decomposition analysis was conducted to assess the contributions of aging, population growth, and epidemiologic changes over the past decades. This study utilized the method developed by Das Gupta to perform the analysis (15). Decomposition analysis is like breaking down a big change into smaller parts to see what contributed to it the most—whether it’s aging, more people, or changes in how diseases are spread. The decomposition method isolates the impact of each factor on changes in disease indicators by holding other variables constant. In simple terms, this method lets us see how much each factor, like population growth or aging, is responsible for the increase or decrease in cancer rates. The resulting effects quantify how much a specific factor, such as aging or population growth, contributes to changes in the overall burden. For example, we can see how much of the change in cancer cases is due to more people getting older, or how much is due to the population growing. Importantly, the combined effects of all factors equal the total observed change in the indicator, providing a clear understanding of the relative influence of each driver on the evolving epidemiology of GBTC. Together, all of these factors add up to explain the overall changes in cancer rates over time, giving us a clear picture of what is driving the trends. In this analysis, “epidemiologic changes” refer to all residual factors beyond demographic shifts, including changes in disease risk profiles, medical advancements in diagnosis and treatment, environmental exposures, migration patterns, socioeconomic development, healthcare accessibility, and global health events such as the COVID-19 pandemic. Epidemiologic changes are everything else—like better treatments, lifestyle changes, environmental factors, and big events like COVID—that can influence cancer rates over time. This category reflects the complex and multifactorial nature of health transitions that influence disease burden over time.

### Cross-country inequality analysis

Health inequality monitoring provides a foundation for evidence-based health planning to address disparities in health outcomes. This study utilized the slope index of inequality and concentration index to evaluate SDI-related inequalities in the incidence, prevalence, DALYs, and deaths of GBTC among adults age 45 years and older (16). The slope index measured absolute disparities by regressing national GBTC rates on an SDI-related relative position scale, while the concentration index assessed relative inequality through Lorenz curve analysis (17).

### Statistics analysis

The incidence, prevalence, DALYs, and deaths were represented as estimates per 100,000 population, accompanied by their 95% CIs, while EAPCs were also presented with their 95% CIs. All procedures for analysis and graphic representation were performed using the statistical computing R Studio (Version 4.3.3 for Windows). A *P*-values < 0.05 was considered statistically significant.

## Results

### Global trends

**Figure 1:**
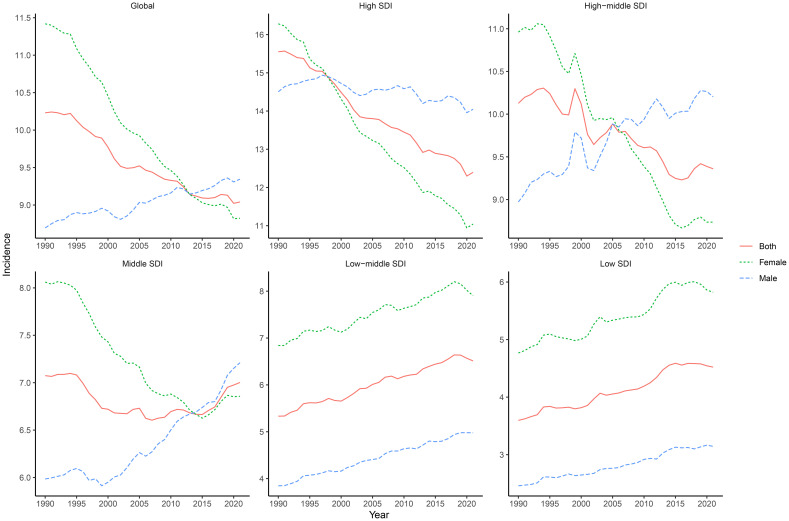
Global and SDI-stratified incidence trends of GBTC from 1990 to 2021. This figure presents the incidence trends of GBTC globally and across different levels of the SDI from 1990 to 2021. The trends are shown separately for both genders, highlighting the variations in GBTC incidence over time and among different SDI groups.

In 2021, GBTC among individuals aged 45 years and older accounted for 209,463 incident cases globally, with an ASIR of 9.04 per 100,000 population and an average annual decrease of 0.45% since 1990 (**Table 1**). Prevalence reached 300,560 cases, with an ASPR of 12.86 per 100,000 and an average annual increase of 0.24% (**Supplementary Table S1**). DALYs were estimated at 3,499,957, with an ASDR of 147.62 per 100,000 and an average annual decrease of 1.080% (**Supplementary Table S2**), while deaths numbered 167,529, with an ASMR of 7.27 per 100,000 and an annual decrease of 0.90% (**Supplementary Table S3**). Notably, ASIR and ASPR decreased among females but increased among males (**Figure 1**, **Supplementary Figure S1**), while ASRs for both DALYs and mortality consistently declined in both genders (**Supplementary Figure S2**, **Supplementary Figure S3**).

**Table 1:** Age standardized incidence rate (ASIR) of gallbladder and biliary tract cancer in 1990 and 2021 and estimated annual percentage change (EAPC) from 1990 to 2021 at the global and regional level.

Category	1990		2021		1990-2021
	Incident cases, (95% CI)	ASIRs per 100 000 (95% CI)	Incident cases, (95% CI)	ASIRs per 100 000 (95% CI)	EAPC, %, (95% CI)
Global	103029.36(91477.77,113416.25)	10.23(9.06,11.25)	209463.27(170963.61,238646.20)	9.04(7.37,10.31)	-0.45(-0.49,-0.42)
SDI					
High	47863.75(43521.87,50670.14)	15.552(14.12,16.48)	77645.79(65329.54,86757.11)	12.40(10.56,13.77)	-0.78(-0.81,-0.74)
High-middle	26335.74(22765.23,28474.18)	10.13(8.75,10.96)	51216.23(39005.90,60240.33)	9.36(7.13,11.01)	-0.36(-0.41,-0.31)
Middle	18287.42(15526.12,23250.43)	7.07(6.01,9.00)	50373.97(40189.14,64378.15)	7.01(5.59,8.96)	-0.12(-0.21,-0.03)
Low-middle	8309.58(6896.21,11786.75)	5.33(4.42,7.56)	24393.31(18917.92,30473.68)	6.51(5.06,8.16)	0.72(0.68,0.76)
Low	2091.15(1608.07,2909.82)	3.596(2.78,5.02)	5677.94(3863.74,7195.22)	4.52(3.07,5.71)	0.844(0.78,0.91)
Regions					
Andean Latin America	921.62(698.33,1138.57)	17.42(13.23,21.51)	2234.47(1630.07,3045.24)	14.12(10.31,19.23)	-0.88(-1.05,-0.71)
Australasia	770.97(691.01,851.68)	11.95(10.67,13.22)	1793.18(1476.32,2096.57)	11.74(9.74,13.67)	-0.050(-0.23,0.13)
Caribbean	394.97(337.54,454.76)	5.72(4.89,6.59)	517.51(432.27,612.17)	3.49(2.92,4.13)	-1.79(-1.91,-1.68)
Central Asia	466.59(407.72,544.20)	3.74(3.26,4.37)	626.40(549.75,713.78)	2.94(2.58,3.35)	-1.27(-1.75,-0.79)
Central Europe	6393.01(5934.33,6760.60)	15.80(14.62,16.74)	6321.67(5649.87,6975.07)	10.00(8.95,11.04)	-1.67(-1.77,-1.61)
Central Latin America	3195.61(3031.10,3340.25)	15.06(14.23,15.77)	5138.28(4513.30,5787.00)	7.60(6.67,8.56)	-2.54(-2.73,-2.36)
Central Sub-Saharan Africa	60.10(39.17,92.74)	1.08(0.71,1.65)	154.28(98.96,229.17)	1.16(0.74,1.74)	0.37(0.24,0.49)
East Asia	16438.09(12476.50,21050.97)	7.64(5.75,9.75)	51185.73(35098.74,66713.17)	8.56(5.86,11.13)	0.41(0.32,0.50)
Eastern Europe	4468.06(4128.61,4853.39)	5.81(5.36,6.31)	6364.67(5754.89,6940.00)	6.48(5.86,7.07)	-0.07(-0.35,0.20)
Eastern Sub-Saharan Africa	696.47(467.95,958.17)	3.67(2.50,5.01)	1371.03(952.12,1853.19)	3.37(2.34,4.53)	-0.40(-0.49,-0.31)
High-income Asia Pacific	18078.78(16144.82,19614.04)	33.90(30.09,36.87)	36500.98(29097.33,42558.52)	23.61(19.29,27.38)	-1.23(-1.28,-1.17)
High-income North America	8751.39(7990.01,9230.93)	8.85(8.13,9.37)	13707.32(12232.31,14686.21)	7.41(6.66,7.93)	-0.60(-0.66,-0.54)
North Africa and Middle East	2043.85(1583.27,2728.01)	4.90(3.80,6.61)	5544.11(4005.61,6917.03)	4.91(3.56,6.14)	0.23(0.10,0.36)
Oceania	15.85(9.22,22.18)	2.20(1.32,3.03)	33.75(22.30,46.13)	1.81(1.21,2.45)	-0.65(-0.69,-0.62)
South Asia	8620.56(6829.77,12305.58)	5.83(4.625,8.358)	30967.332(21560.639,37095.915)	7.979(5.573,9.578)	1.054(0.998,1.109)
Southeast Asia	3816.65(2751.30,5036.15)	5.90(4.27,7.81)	11514.51(8077.49,15290.36)	6.83(4.79,9.07)	0.31(0.23,0.39)
Southern Latin America	3765.28(3392.58,4137.01)	30.21(27.13,33.25)	4305.59(3756.63,4844.69)	17.85(15.59,20.07)	-1.78(-1.86,-1.70)
Southern Sub-Saharan Africa	160.99(115.15,218.73)	2.36(1.67,3.20)	432.78(294.59,522.26)	2.94(1.98,3.54)	0.89(0.73,1.05)
Tropical Latin America	2552.47(2364.71,2705.51)	11.03(10.12,11.74)	5555.70(5023.66,5968.92)	7.96(7.17,8.57)	-1.20(-1.35,-1.05)
Western Europe	21383.43(19442.54,22856.36)	13.00(11.83,13.89)	25106.39(21652.65,27557.10)	9.10(7.98,9.92)	-1.19(-1.31,-1.07)
Western Sub-Saharan Africa	34.62(26.82,49.22)	0.156(0.12,0.22)	87.59(57.47,112.04)	0.19(0.12,0.24)	1.23(0.86,1.60)

ASIR, age standardized incidence rate; EAPC, estimated annual percentage change; SDI, socio-demographic index; 95% CI, 95% confidence interval.

In 2021, across different SDI regions, the high SDI region had the highest ASIR, ASPR, and ASMR, while the low SDI region had the lowest for these metrics (**Table 1**, **Supplementary Table S1**, and **Supplementary Table S3**). However, the ASDR was highest in the low-middle SDI region and lowest in the low SDI region (**Supplementary Table S2**). From 1990 to 2021, the low SDI region experienced the greatest increase in ASIR, ASPR, ASDR, and ASMR, while the high SDI region showed the largest decrease in ASIR and ASDR (**Table 1**, **Supplementary Table S2**, and **Supplementary Table S3**). ASPR showed the smallest increase in the high SDI region (**Supplementary Table S1**).

**Figure 2:**
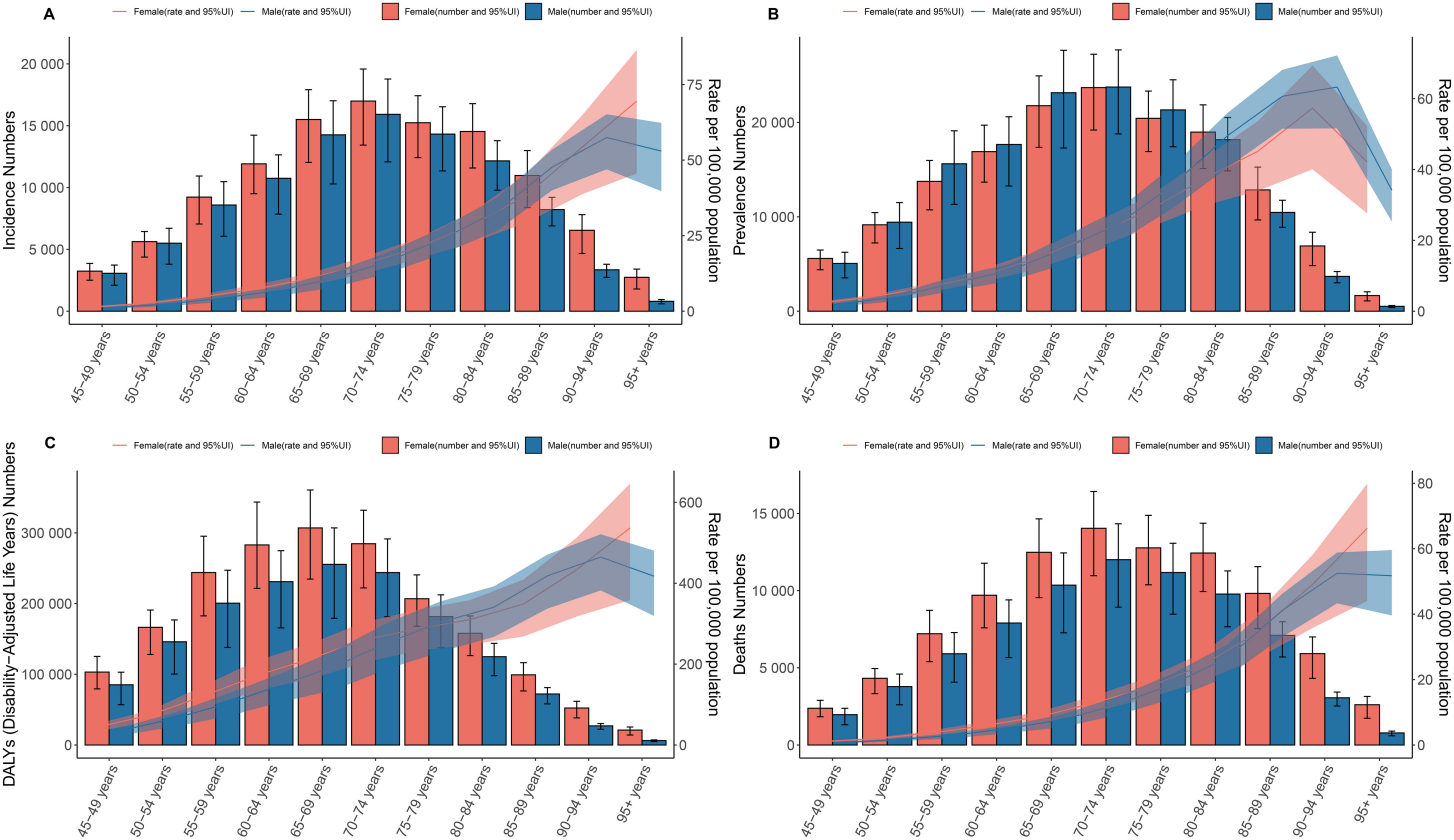
Age and sex patterns of GBTC at the global level. Depicting the incidence, prevalence, DALYs, and deaths related to GBTC by age and sex. **(A)** Incidence. **(B)** Prevalence. **(C)** DALYs. **(D)** Deaths. Both the rates and numbers are presented, showing the differences in GBTC occurrence and impact between males and females across different age cohorts globally.

In 2021, the burden of GBTC among individuals aged 45 years and older exhibited distinct age-related and gender-specific patterns. Incident and prevalent cases increased with age, peaking in the 70–74 age group before decreasing, while incidence and prevalence rates increased steadily with age but started to decrease after the 90–94 age group (**Figures 2A, B**). DALYs showed a similar trend, peaking in the 65–69 age group and then decreasing, with females consistently having higher DALYs than males across all age groups. For males, the DALY rate increased with age but began to decrease after the 90–94 age group (**Figure 2C**). Deaths also increased with age, peaking in the 70–74 age group before decreasing, with females consistently having higher death counts than males. The mortality rate for males increased with age and similarly decreased after the 90–94 age group (**Figure 2D**).

### Regional trends

In 2021, among the 21 GBD regions, East Asia, High-income Asia Pacific, and South Asia had the highest number of prevalent cases in 2021 (**Table 1**). East Asia, High-income Asia Pacific, and Western Europe had the highest numbers of prevalent cases in 2021 (**Supplementary Table S1**). East Asia, South Asia, and High-income Asia Pacific having the highest number of DALYs and deaths cases due to GBTC among individuals aged 45 years and older in 2021 (**Supplementary Tables S2**-**S3**).

In 2021, the burden of GBTC showed notable disparities across the 21 GBD regions. High-income Asia Pacific consistently reported the highest rates, including ASIR (23.61), ASPR (35.58), ASDR (305.56), and ASMR (17.35), followed by Southern Latin America and Andean Latin America, which also ranked among the top three for these metrics. Conversely, the lowest rates were observed in Western Sub-Saharan Africa, Central Sub-Saharan Africa, and Oceania, where ASIR and ASPR were below 2 per 100,000, and ASDR and ASMR were significantly lower compared to other regions. For instance, Western Sub-Saharan Africa had an ASIR of 0.19, ASPR of 0.18, ASDR of 4.32, and ASMR of 0.21, marking the lowest levels globally (**Tables 1**, **S1**-**S3**). **Supplementary Figures S4**-**S7** illustrate these trends by sex, showing consistent patterns across all regions.

During the period from 1990 to 2021, we found that the EAPC of ASIR was highest in Western Sub-Saharan Africa (1.23%), South Asia (1.05%), and Southern Sub-Saharan Africa (0.89%), while Central Latin America (-2.54%), Caribbean (-1.79%), and Southern Latin America (-1.78%) had the highest decrease (**Table 1**). In the same period, the EAPC of ASPR, ASDR and ASMR showed a similar ranking of highest and lowest values (**Supplementary Tables S1**-**S3**). **Supplementary Figures S8**-**S11** show the EAPC of ASIR, ASPR, ASDR and ASMR by sex for all regions in the 21 regions in GBD 2021.

**Figure 3:**
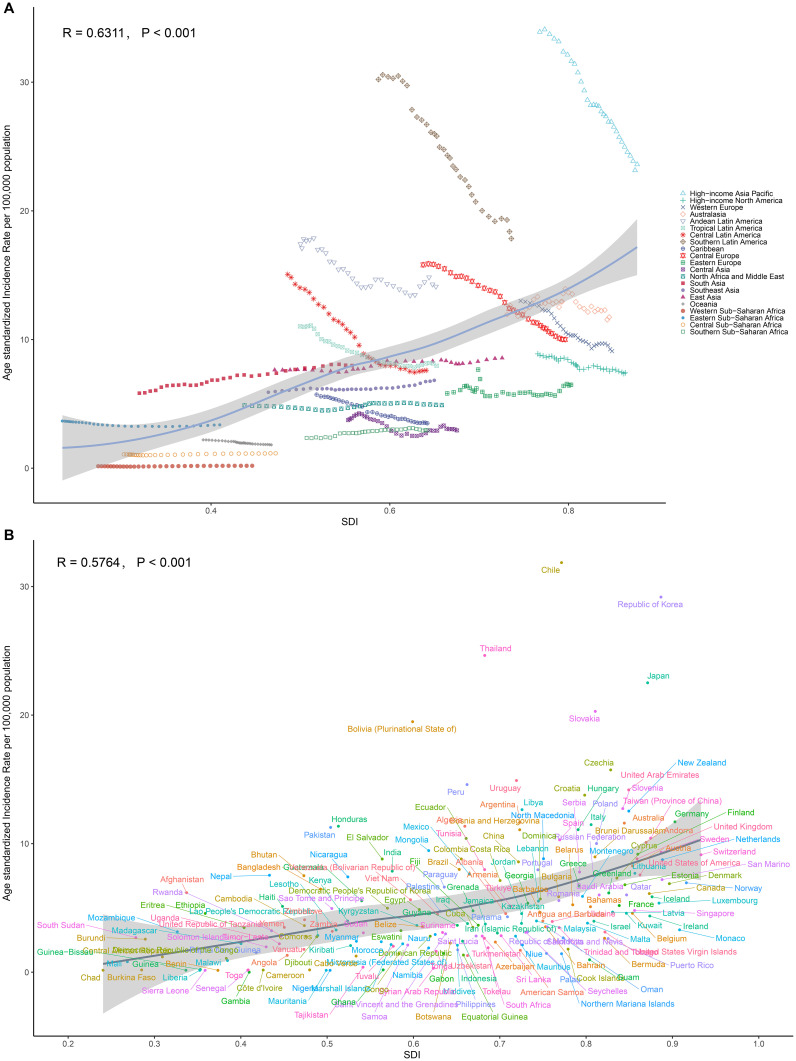
Relationships between SDI and ASRs of GBTC. **(A)** SDI and ASIR among different regions. **(B)** SDI and ASIR among different countries. The scatter plots show the trends and correlations, with regression lines and corresponding R and P values, indicating the strength and significance of the relationships among different regions or nations.

Regionally, a positive relationship was observed between the SDI and the ASIR of GBTC since 1990. Initially, as the SDI increased, the ASIR showed a upward trend (**Figure 3A**). Notably, High-income Asia Pacific exhibited significantly higher ASIR than expected based on their SDI during this period, whereas Western Sub-Saharan Africa consistently had much lower-than-expected ASPR (**Figure 3A**). Similar patterns were found for ASPR, ASDR, and ASMR) when analyzed in relation to SDI (**Supplementary Figures S12**-**S14**).

### National trends

**Figure 4:**
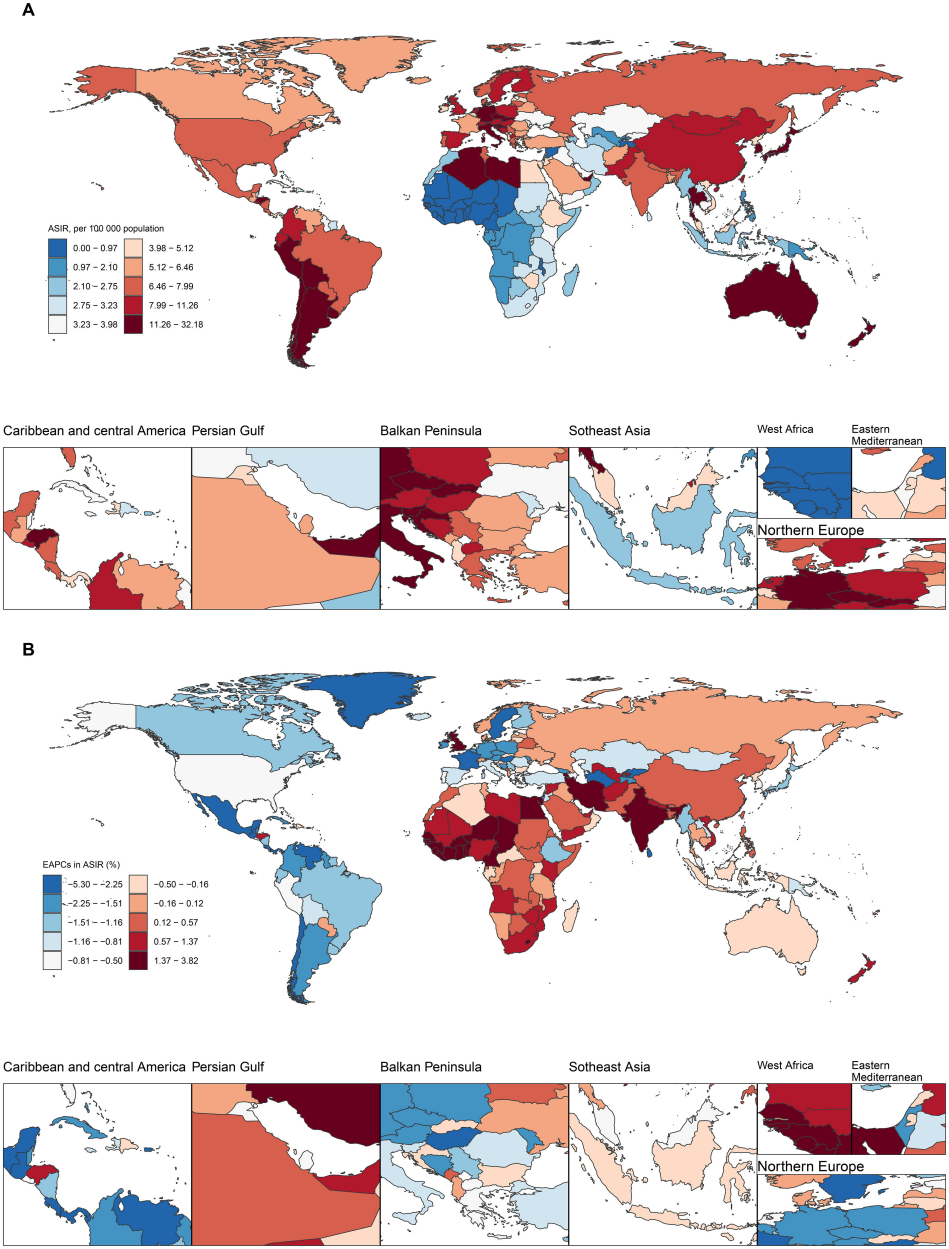
Maps of ASIR of GBTC in 2021 and EAPC in ASIR from 1990 to 2021 in 204 countries and territories.**(A)** Global distribution of ASPR in units per 100,000 population. **(B)** Average annual percentage change in ASPR. Illustrating the global geographical distribution of ASIR, of GBTC in 2021. The maps are color-coded based on different rate ranges, providing a visual representation of the regional disparities in GBTC burden.

In 2021, the burden of GBTC varied significantly across countries. The highest rates for ASIR (31.86), ASPR (43.40), ASDR (583.38), and ASMR (27.10) were all observed in Chile, followed by Republic of Korea (ASIR 29.18, ASPR 43.40, ASMR 21.56) and Thailand (ASIR 24.64, ASDR 507.40, ASMR 22.50). Conversely, the lowest rates for all four metrics were found in Gambia (ASIR 0.01, ASPR 0.01, ASDR 0.01, ASMR 0.01), Niger (ASIR 0.13, ASPR 0.12, ASDR 2.91, ASMR 0.15), and Benin (ASIR 0.14, ASPR 0.13, ASDR 3.08) (**Figures 4A**, **S15A**, **S16A**, **S17A**, **Supplementary Tables S4**).

From 1990 to 2021, the largest increases in ASIR, ASPR, ASDR, and ASMR were consistently seen in Cabo Verde (ASIR 3.79%, ASPR 3.87%, ASDR 3.49%, ASMR 3.71%), Armenia (ASIR 2.97%, ASPR 3.12%, ASDR 2.72%, ASMR 2.88%), and Lesotho (ASIR 2.90%, ASDR 2.96%, ASMR 2.89%). In contrast, the largest decreases were observed in Turkmenistan (ASIR -5.25%, ASPR -5.16%, ASDR -5.25%, ASMR -5.29%), Sri Lanka (ASIR -5.03%, ASPR -4.51%, ASDR -5.51%, ASMR -5.44%), and Guatemala (ASIR -4.36%, ASPR -4.14%, ASDR -4.39%, ASMR -4.46%) (**Figures 4B**, **S15B**, **S16B**, **S17B**, **Supplementary Tables S4**).

At the national level in 2021, the ASIR of GBTC generally increased as the SDI increased. The Chile showed a much higher than expected burden, whereas Gambia demonstrated a considerably lower than expected burden (**Figure 3B**). Similar national-level patterns were observed for ASPR, ASDR, and ASMR in relation to SDI (**Supplementary Figures S12B**, **S13B**, **S14B**).

### Risk factor

**Figure 5:**
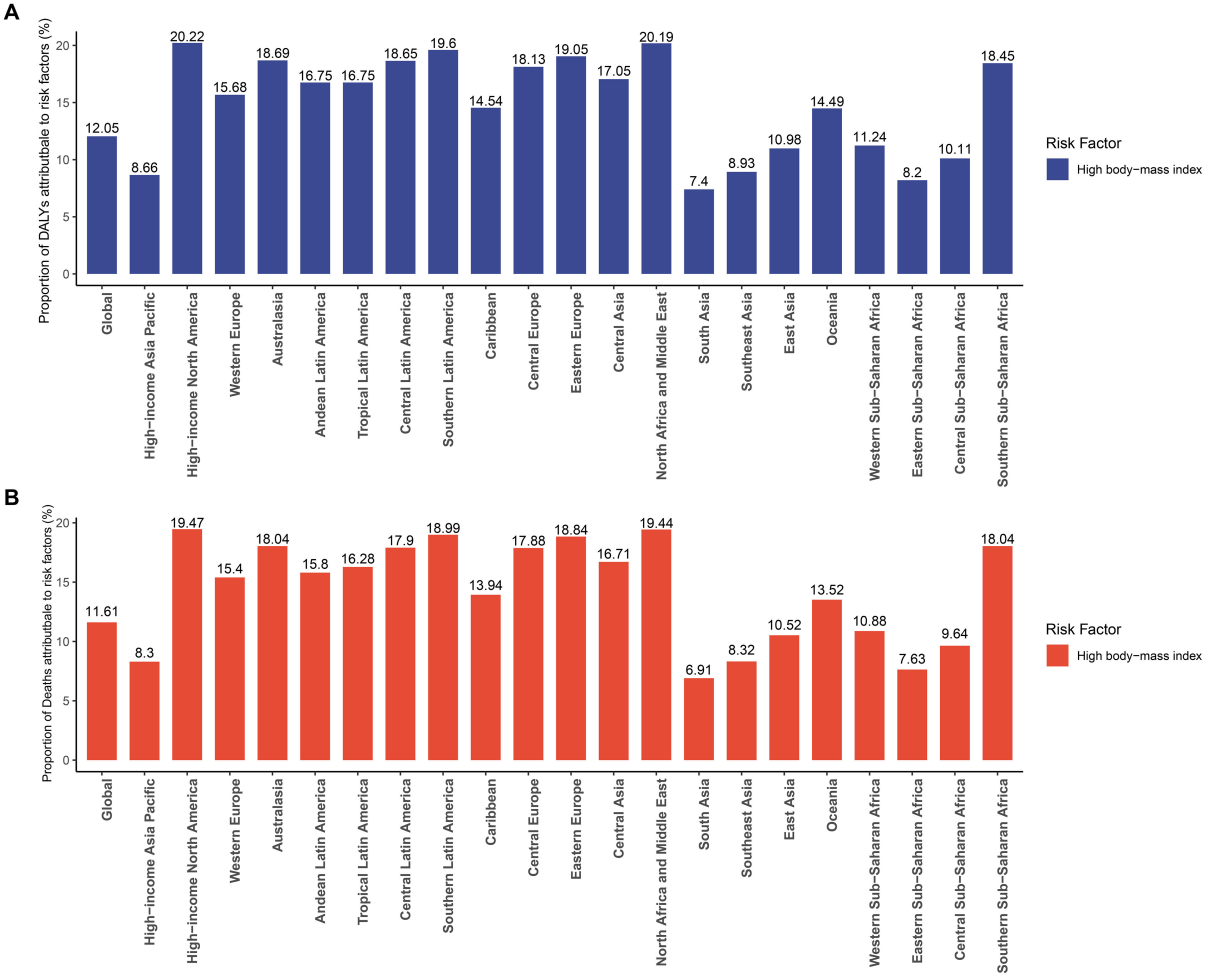
Percentage of **(A)** DALYs and **(B)** deaths due to high body-mass index for the Global Burden of Disease regions in 2021.

The contribution of high body-mass index to DALYs and deaths caused by GBTC varies across Global Burden of Disease regions. Globally, 12.05% of DALYs and 1.61% of deaths from GBTC were attributable to high BMI in the GBD framework (**Supplementary Figure S18**). Regionally, High-income North America had the highest proportion of DALYs (20.22%) attributable to high BMI (**Figure 5A**), as well as the highest proportion of deaths (19.47%) (**Figure 5B**). The distribution of DALYs and deaths attributable to high BMI across different age groups is shown in **Supplementary Figures S19**. For the contribution of high BMI to DALYs and deaths, we found that the 50–54 age group had the highest proportions, at 13.08% and 13.06%, respectively.

### Frontier analysis

**Figure 6:**
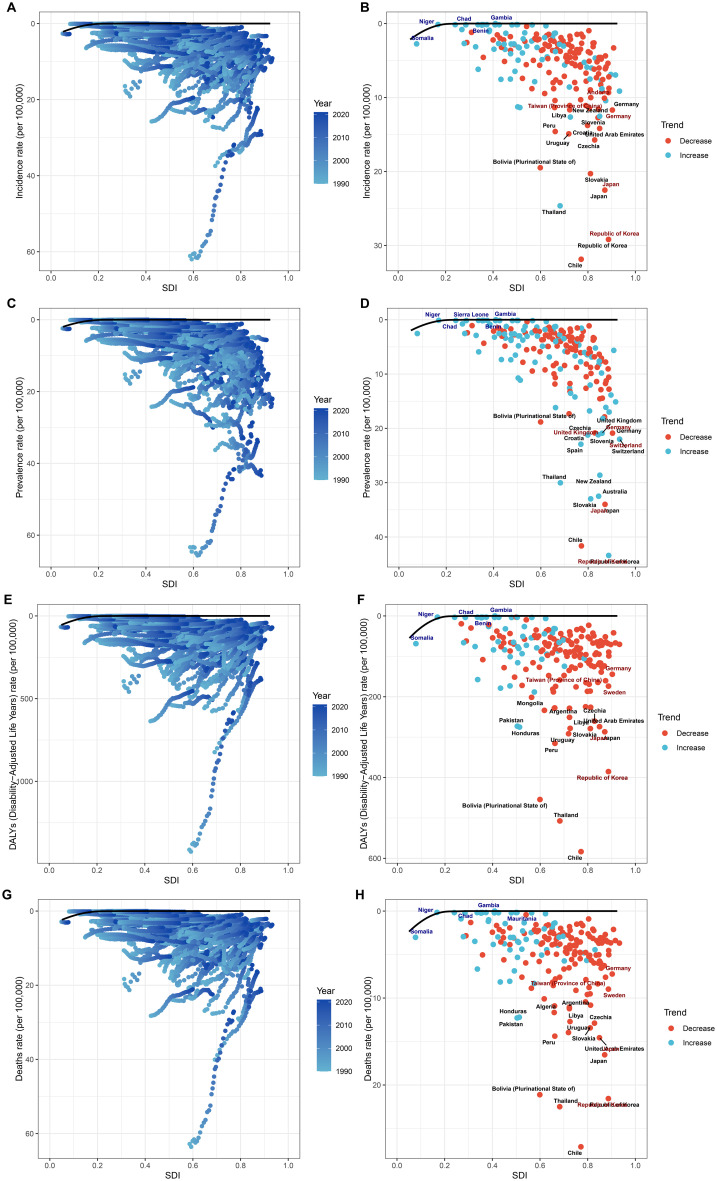
Frontier analysis of GBTC burden in relation to SDI. Illustrating the frontier analysis results of GBTC burden in relation to the SDI. **(A)** SDI and ASIR among different regions. **(B)** SDI and ASIR among different countries. **(C)** SDI and ASPR among different regions. **(D)** SDI and ASPR among different countries. **(E)** SDI and ASDR among different regions. **(F)** SDI and ASDR among different countries. **(G)** SDI and ASMR among different regions. **(H)** SDI and ASMR among different countries. The figures show the differences between the observed and achievable minimum ASRs (incidence, prevalence, DALYs, and mortality rates) across various countries and regions, highlighting the potential for improvement in GBTC burden management.

Using data from 1990 to 2021, we performed a frontier analysis of ASIR, ASPR, ASDR, and ASMR in relation to the SDI to assess the potential for reducing the burden of GBTC while accounting for national and regional development levels (**Figure 6**, **Supplementary Tables S5**-**S8**). The frontier curve represents the lowest (best-performing) ASR for a given SDI, while the distance from the curve, termed the “effective difference,” quantifies the gap between a country or region’s observed ASR and the optimal ASR under ideal conditions. In 2021, the effective difference for ASIR was calculated for each country and region based on their SDI (**Figure 6B**). Overall, the effective difference tended to decrease as the SDI increased, with variance stabilizing at higher SDI levels. Interestingly, frontier countries and regions with lower SDI, such as Somalia, Niger, Chad, Benin, and Gambia, performed relatively well despite limited resources. Similar patterns were observed in the frontier analyses for ASPR (**Figures 6C, D**), ASDR (**Figures 6E, F**), and ASMR (**Figures 6G, H**), further emphasizing the relationship between SDI and the potential for reducing the burden of GBTC.

### Decomposition analysis

**Figure 7:**
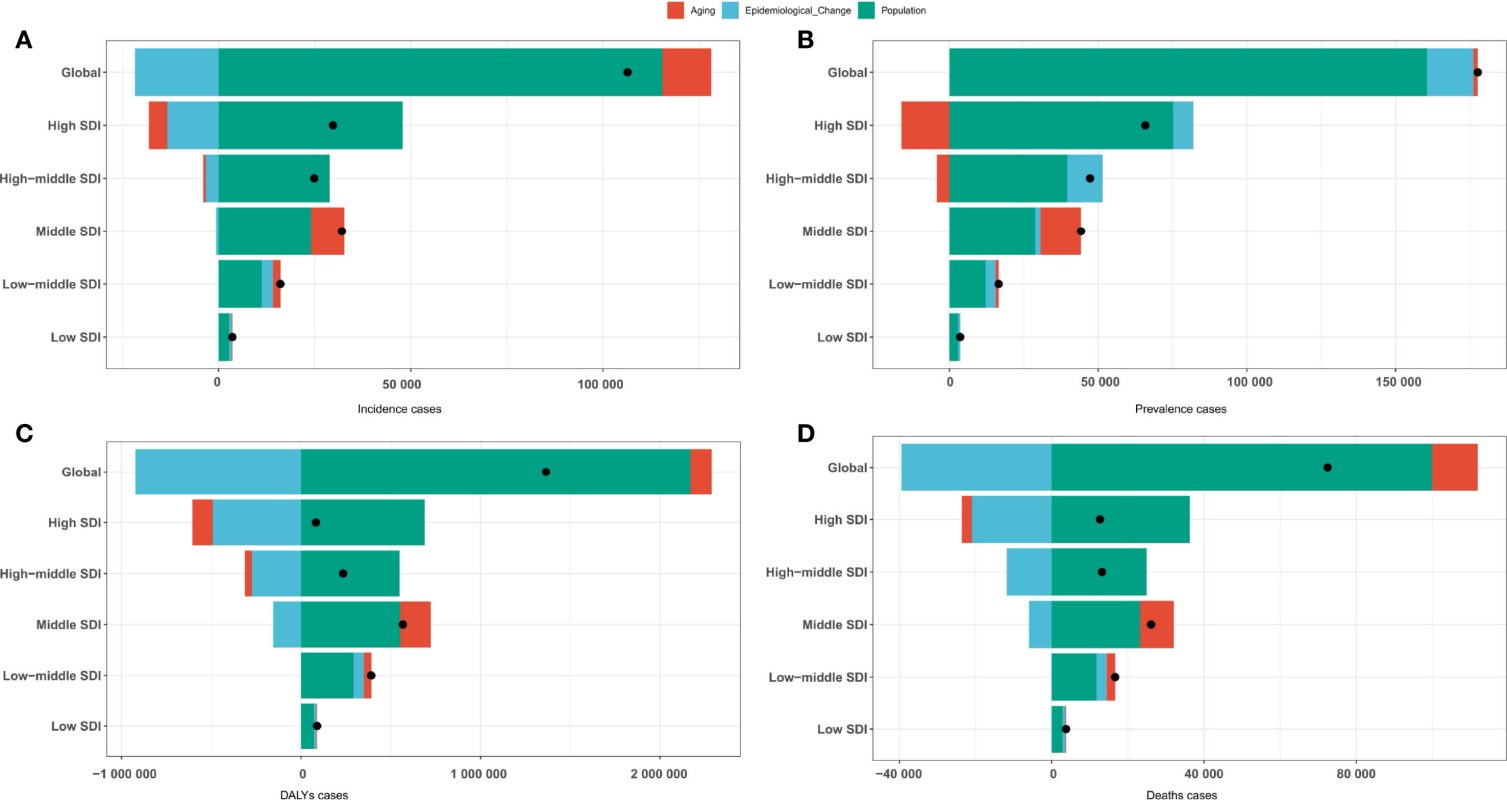
Decomposition analysis by population growth, epidemiological changes, and aging of GBTC for the Global Burden of Disease regions. **(A)** Incident cases. **(B)** Prevalent cases. **(C)** DALYs cases. **(D)** Deaths cases.

Globally, from 1990 to 2021, population growth, epidemiological changes, and aging contributed 11.94%, 108.51%, and -20.45%, respectively, to the increase in GBTC incident cases (**Figure 7A** and **Supplementary Table S9**). Across SDI regions, the contribution of population growth to the overall number of incident cases was most significant in high SDI regions and smallest in low SDI regions. A similar pattern was observed for prevalent cases, DALYs, and deaths (**Figures 7B-D** and **Supplementary Tables S10**-**S12**), highlighting the consistent impact of population growth and epidemiological shifts on the global burden of this cancer type.

### Cross-country analysis

**Figure 8:**
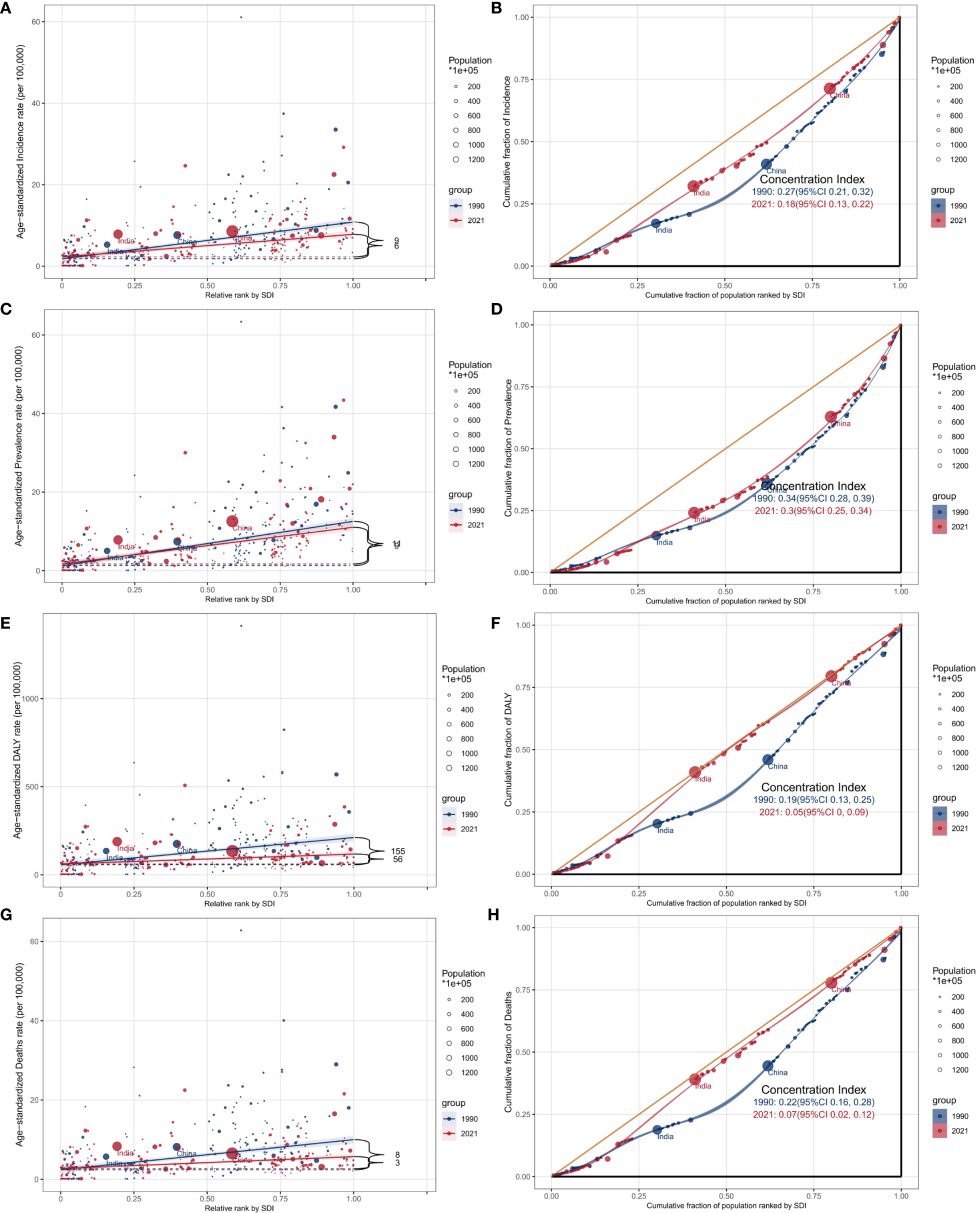
SDI-related health inequality analysis of GBTC from 1990 to 2021 by 204 countries and territories. **(A)** Health inequality regression curves for age-standardized incidence rate; **(B)** concentration curves for age-standardized incidence rate; **(C)** Health inequality regression curves for age-standardized prevalence rate; **(D)** concentration curves for age-standardized prevalence rate; **(E)** Health inequality regression curves for age-standardized DALYs rate; **(F)** concentration curves for age-standardized DALYs rate. **(G)** Health inequality regression curves for age-standardized incidence rate; **(H)** concentration curves for age-standardized incidence rate.

Significant inequalities in the burden of GBTC were observed across countries with different SDI levels, and these disparities have increased over time (**Figures 8A, B**). Overall, countries with higher SDI had a higher concentration of GBTC incidence. The slope index of inequality showed a slight reduction in the prevalence gap between the highest and lowest SDI countries, from 9 per 100,000 in 1990 to 6 in 2021. Similarly, the concentration index, reflecting relative inequalities, decreased from 0.27 in 1990 to 0.18 in 2021, indicating a modest narrowing of inequalities. These trends were consistent for ASPR (**Figures 8C, D**), ASDR (**Figures 8E, F**), and ASMR (**Figures 8G, H**), which also demonstrated uneven distributions of the GBTC burden across countries with varying SDI levels. In summary, while inequalities persist, there has been some narrowing of the gap in certain aspects, although in other areas, disparities remain largely unchanged.

## Discussion

### Principal findings

From 1990 to 2021, the global burden of GBTC among adults age 45 years and older showed notable trends, with a slight 0.453% annual decrease in incidence and a 0.240% annual increase in prevalence. Despite a global decline in deaths and DALYs, the incidence and prevalence rates were higher in males. Regional disparities were evident, with high-SDI regions experiencing the highest rates of incidence, prevalence, DALYs, and mortality, while low-SDI regions had the lowest burden. The low-SDI regions, however, experienced the largest increases in cancer rates. A significant increase in GBTC burden was observed in countries like Cabo Verde, Armenia, and Lesotho, whereas nations like Turkmenistan and Sri Lanka saw the largest decreases. The global contribution of high BMI to DALYs and deaths was particularly high in high-income regions like North America, with the 50-54 age group being most affected. Projections suggest a continued decrease in age-standardized rates in high-SDI regions, but the persistent disparities in the global burden, particularly in low-SDI countries, highlight the need for tailored public health strategies to reduce inequalities and improve healthcare access.

### Comparison with other studies

Previous studies have shown that the ASR of mortality and DALYs of GBTC slightly decreased from 1990 to 2021 (1). The results of this study show that in 2021, the incidence of GBTC among individuals aged 45 and above globally showed a slight decline. The incidence of GBTC in this study was consistent with the incidence trend of the entire population in previous studies. This trend may be attributed to improvements in public health measures and early cancer screening. A study on people over 55 years old found that the incidence and number of cases of GBTC increased, which was different from the results of this study (3). However, the period analyzed in this study was from 2010 to 2021, while in this study it was from 1990 to 2021, and the population included in this study was people over 45 years old. By comparing the two studies, it seems that we can infer that in recent years, the disease burden of GBTC in older age groups has shown an increasing trend. Therefore, in the future, a period cohort study on the disease burden of GBTC will be conducted to provide a theoretical basis for precise prevention and control. However, the incidence and mortality rates remain higher in men, consistent with previous research findings on gender differences. Men are more susceptible to this type of cancer due to physiological, lifestyle, and possibly genetic factors (18). While smoking and alcohol consumption show epidemiological associations with GBTC risk, their causal role remains incompletely elucidated (19). Additionally, regions with a low SDI show lower incidence rates, which may be linked to limited healthcare resources, lower levels of diagnosis, and fewer cancer screening activities in these areas (20). Low SDI regions often face greater health challenges, particularly in the early detection and treatment of GBTC. The relatively low burden in these areas may not fully reflect the true epidemiological situation, partly due to underreporting of data and insufficient screening (21). GBTC incidence and mortality rates are generally lower in low SDI regions, but the actual burden may be higher, as diagnostic and reporting systems in these areas are often inadequate (22).

This trend of incidence for GBTC is likely closely related to the dietary habits in these areas (23). Previous studies found that dietary factors increase the risk of gallstones formation, which may increase the risk of GBTC (24). In addition, genetic susceptibility is an important factor that cannot be ignored. Studies showed that East Asian populations have certain genetic characteristics that make them more susceptible to GBTC infection, which may lead to a higher incidence rate in these areas (25). In the high-income Asia-Pacific regions, despite advanced medical care and cancer screening systems, the incidence of GBTC is still high due to lifestyle factors (26). In contrast, Sub-Saharan Africa and Oceania have lower GBTC incidence. This phenomenon is mainly attributed to the relatively low cancer screening rates and diagnostic levels in these regions, resulting in lower early detection and insufficiently reflected of cancer burden (27, 28). Additionally, there are significant differences in dietary patterns between Sub-Saharan Africa and Oceania and East Asia. The lower-fat diets and lower prevalence of gallstones in these regions may also help to reduce the incidence of GBTC to some extent (7).

A high-fat and low fiber diet may increase the risk of gallstones and cholecystitis, both of which are important risk factors for gallbladder cancer (29). In addition, while Chile’s healthcare system is relatively advanced in certain areas, there are still shortcomings in early screening and diagnosis of GBTC, which may lead to an increased in the country’s cancer (3). In contrast, the incidence of GBTC increased significantly in Cape Verde, Armenia, and Lesotho. This may be related to changes in health management, lifestyle shifts, and improvements in cancer testing in these countries. In low-middle SDI countries such as Cape Verde and Lesotho, recent improvements in public health systems and cancer screening capabilities may have increased early cancer diagnosis, resulting in an increase in reported cancer cases (30). Additionally, the increasing burden of cancer in Armenia may be associated with population aging and westernization of dietary habits. Western diets, such as high-fat and high salt diets, have been identified as risk factors for GBTC. The changes of lifestyle in Armenia in recent years may be the reason for the rise of its incidence (31).

High BMI is an important risk factor for GBTC in the GBD framework. Previous studies on the entire population have shown that the burden of GBTC attributed to high BMI is reduced by 7 worldwide. The results of this study show that the disease burden of GBTC remains relatively high in high-income North America and among people aged 50-54. According to the “World Obesity Atlas 2024”, by 2020, the number of overweight and obese people in high-income countries was 314.39 million and 271.9 million respectively, which was more than seven times that of low-income countries (32). With the development of the social economy, in recent years, the lifestyle in countries with high SDI has undergone tremendous changes. More people choose to eat fast food, adopt more convenient and faster travel methods, and use electronic devices more frequently. All these factors will lead to weight gain. Though both cholesterol gallstones and porcelain gallbladder have been associated with gallbladder cancer; however, the causal relationship remains unclear due to complex confounding factors. Specifically, the evidence for porcelain gallbladder is limited by diagnostic heterogeneity and its pathological rarity (23). Additionally, the dietary patterns in North America characterized by high calorie and high-fat food intake, as well as a sedentary lifestyle, has led to a generally high BMI among this population, further exacerbating the burden of GBTC (33). Particularly in the 50-54 age group, BMI contributes the most, which may be related to more relaxed weight management and metabolic changes in this population. Studies have shown that as individuals age, those with higher BMI face significantly increased metabolic risks and a higher likelihood of developing cancer (34). Furthermore, early interventions for obesity and lifestyle changes can significantly reduce the cancer burden in this age group, especially in high-income countries where resources for health management and disease prevention are more accessible (35).

Countries with high SDI demonstrate greater potential in reducing the burden of cancer, mainly due to their stronger medical resources, advanced screening technologies, and more comprehensive health management systems. In high-income countries, the widespread adoption of early diagnosis and treatment greatly reduces cancer mortality and long-term burden. Countries with high SDI are better able to address the challenges of cancer by implementing effective public health policies, cancer prevention measures, and advancements in medical technology to reduce the burden of disease and increase survival rates (36). Although low-SDI countries bear a heavier cancer burden, countries such as Somalia, Niger, and Gambia have also shown potential for improvement. This is partly due to the recent progress made by these countries in health infrastructure and disease prevention. By introducing vaccination programs plans, improving screening coverage and strengthening basic healthcare services, low-SDI countries can effectively control the incidence and mortality of cancer (37). In recent years, Niger has made some progress in strengthening basic healthcare and health education, providing potential opportunities to reduce the cancer burden. High-SDI countries can significantly reduce their cancer burden through technological advancements and public health interventions, while low-SDI countries also can reduce the cancer burden and improve their health levels as resources gradually improve. Our findings necessitate context-specific interventions: High-SDI regions should prioritize metabolic risk control, particularly BMI reduction programs targeting adults aged 50-54 years, which could prevent GBTC deaths. Middle-SDI regions like Chile require early detection enhancement. We hypothesize that mandatory cholecystectomy for gallstone patients under 50 may be beneficial, but further research is needed to confirm this approach. Low-SDI regions must address diagnostic gaps by exploring the potential of task-shifting ultrasound screening to primary care workers. We suggest integrating GBTC screening into national NCD programs and propose developing SDI-stratified treatment guidelines along with establishing international referral networks for complex cases. In addition, for people aged 50-54 with a high BMI, it could be valuable to explore multi-party coordinated intervention approaches: community health management centers may collaborate with medical insurance support to provide comprehensive “weight loss + metabolic risk” clinics, combining nutritional guidance, exercise prescriptions, and behavioral interventions, with the goal of reducing BMI by ≥5% within 6 months, while integrating blood pressure, blood lipid, and blood sugar monitoring to improve long-term health prognosis (38).

Population growth and epidemiological changes are the main reasons for the increase in global GBTC cases. As the global population continues to rise, particularly in middle- and low-income countries, cancer incidence also increases. These regions often face higher cancer risk factors, such as poor diet, and limited health interventions, leading to a higher cancer burden (39). Additionally, changes in epidemiological, such as disease patterns, environmental factors, and the widespread adoption of cancer screening techniques, have also contributed to the increase in cancer cases. Especially, with the increase in early diagnosis rates of cancer, more cases are being discovered and reported, further exacerbating the global cancer burden (40). However, population aging has had a negative impact on the global increase in cancer cases. With the aging of the global population, especially in developed countries, the elderly population becomes the primary group with a high incidence of GBTC, partly due to long-term exposure to environmental carcinogens and immune system decline during the aging process (31).

The incidence rate of cancer is significantly different between high-SDI and low-SDI countries. Although these gaps have slightly narrowed in recent years, the cancer burden is still mainly concentrated in high-SDI countries. High-income countries typically have more advanced medical facilities, higher health awareness, and more comprehensive early screening and treatment systems, which enable them to effectively control the occurrence of cancer and improve survival rates. However, lifestyle factors in these countries, such as high-fat diets, lack of physical activity, and high smoking rates, continue to increase the burden of cancer (41). Additionally, high-SDI countries often face the challenge of population aging, with the elderly group at higher risk for GBTC, resulting in a sustained high burden in these countries. On the contrary, although the cancer mortality rate in countries with low SDI is high, their lower incidence rates may partly be due to lower cancer screening rates, leading to many cases being undiagnosed and undetected in the early stages (20). Moreover, the scarcity of medical resources and inadequate public health systems in low-income countries lead to insufficient cancer treatment and interventions, resulting in higher cancer mortality rates in these countries. Nonetheless, with the improvement of medical conditions in low SDI countries and the increase of global health resources, it is expected that the burden of cancer will gradually decrease. Therefore, reducing the cancer burden disparity between high- and low-SDI countries requires more global health resources and health interventions targeted at low-income countries.

### Strengths and limitations of the current study

This study provides a thorough examination of the global burden of GBTC in individuals aged 45 years and older, utilizing data from the GBD 2021. It offers detailed insights into trends in incidence, prevalence, DALYs, and mortality at global, regional, and national levels. By using age-standardized rates and incorporating the SDI, the study effectively highlights health disparities and provides projections for future trends, offering valuable guidance for public health policy and resource allocation. Additionally, the inclusion of data across multiple time points strengthens the understanding of long-term trends and regional variations in disease burden. However, there are several limitations. Data gaps and variations in case definitions within the GBD methodology may introduce bias, and the use of confidence intervals rather than uncertainty intervals (UI) may require cautious interpretation. It is important to note that the findings are based on modeled estimates from the GBD framework, not direct primary data. These estimates may be subject to model limitations and should be interpreted as approximations. The availability of limited data from low- and middle-income countries (LMICs) could reduce the accuracy of estimates, as healthcare quality and socio-economic factors may lead to underreporting of cases. This study focused primarily on demographic and metabolic risk factors, but tumor microenvironmental characteristics are also worthy of attention. Previous studies have shown that high lymphatic vessel density is closely associated with lymph node metastasis and poor prognosis in hepatobiliary malignancies (42). This phenomenon may partially explain why the mortality rate of GBTC remains high in high SDI areas with relatively superior medical resources. Analysis of tumor environmental characteristics may require more in-depth analysis in future studies. Furthermore, the lack of subnational data restricts the ability to examine intra-country disparities in disease burden. It is also important to note the ecological fallacy—country-level data cannot directly explain individual-level risk or outcomes, and conclusions about individual behavior and disease risk should be made cautiously based on this aggregated data. Despite these limitations, the study provides the most current estimates of the global burden of GBTC, forming a crucial basis for targeted public health interventions and policy formulation.

## Conclusions

This study, utilizing GBD 2021 data, highlights the significant burden of GBTC among individuals aged 45 years and older. While global trends indicate a decline in incidence, mortality, and DALYs, the disease burden remains disproportionately high in high-SDI regions, particularly in East Asia and High-income Asia Pacific. Additionally, low-SDI regions, although showing the lowest rates, have experienced the largest increases in disease burden over time, pointing to a widening gap in health outcomes. Despite these disparities, continued global health efforts, improvements in cancer prevention, early detection, and treatment, along with targeted public health policies, offer promising avenues to reduce the future burden of GBTC. To achieve further progress, it is essential to prioritize resource allocation and interventions aimed at both high- and low-SDI regions, with a focus on reducing inequalities in cancer care.

## Data Availability

The original contributions presented in the study are included in the article/**Supplementary Material**. Further inquiries can be directed to the corresponding author.

## Supplementary material

Supplementary Figure 1 Global and SDI-stratified ASPR (Age-Standardized Prevalence Rate) trends of GBTC from 1990 to 2021.

Supplementary Figure 2 Global and SDI-stratified ASDR (Age-Standardized Disability Rate) trends of GBTC from 1990 to 2021.

Supplementary Figure 3 Global and SDI-stratified ASMR (Age-Standardized Mortality Rate) trends of GBTC from 1990 to 2021.

Supplementary Figure 4 Sex difference in ASIR of GBTC for the Global burden of disease regions in 2021.

Supplementary Figure 5 Sex difference in ASPR of GBTC for the Global burden of disease regions in 2021.

Supplementary Figure 6 Sex difference in ASDR of GBTC for the Global burden of disease regions in 2021.

Supplementary Figure 7 Sex difference in ASMR of GBTC for the Global burden of disease regions in 2021.

Supplementary Figure 8 EAPC of ASIR for the Global Burden of Disease regions from 1990 to 2021.

Supplementary Figure 9 EAPC of ASPR for the Global Burden of Disease regions from 1990 to 2021.

Supplementary Figure 10 EAPC of ASDR for the Global Burden of Disease regions from 1990 to 2021.

Supplementary Figure 11 EAPC of ASMR for the Global Burden of Disease regions from 1990 to 2021.

Supplementary Figure 12 Relationships between SDI and ASPR of GBTC. (A) SDI and ASPR among different regions. (B) SDI and ASPR among different countries.

Supplementary Figure 13 Relationships between SDI and ASDR of GBTC. (A) SDI and ASDR among different regions. (B) SDI and ASDR among different countries.

Supplementary Figure 14 Relationships between SDI and ASMR of GBTC. (A) SDI and ASMR among different regions. (B) SDI and ASMR among different countries.

Supplementary Figure 15 Maps of ASPR of GBTC in 2021 and EAPC in ASPR from 1990 to 2021 in 204 countries and territories. (A) Global distribution of ASPR in units per 100,000 population. (B) Average annual percentage change in ASPR.

Supplementary Figure 16 Maps of ASDR of GBTC in 2021 and EAPC in ASDR from 1990 to 2021 in 204 countries and territories. (A) Global distribution of ASDR in units per 100,000 population. (B) Average annual percentage change in ASDR.

Supplementary Figure 17 Maps of ASMR of GBTC in 2021 and EAPC in ASMR from 1990 to 2021 in 204 countries and territories. (A) Global distribution of ASMR in units per 100,000 population. (B) Average annual percentage change in ASMR.

Supplementary Figure 18 Percentage of (A) DALYs and (B) deaths due to high body-mass index for the SDI regions in 2021.

Supplementary Figure 19 Percentage of (A) DALYs and (B) deaths due to high body-mass index for the difference age groups.

Supplementary Table 1 Age standardized prevalence rates (ASPR) of gallbladder and biliary tract cancer in 1990 and 2021, and estimated annual percentage change (EAPC) from 1990 to 2021 at the global and regional.

Supplementary Table 2 Age standardized DALYs rate (ASDR) of gallbladder and biliary tract cancer in 1990 and 2021, and estimated annual percentage change (EAPC) from 1990 to 2021 at the global and regional level.

Supplementary Table 3 Age standardized deaths rate (ASMR) of gallbladder and biliary tract cancer in 1990 and 2021, and estimated annual percentage change (EAPC) from 1990 to 2021 at the global and regional level.

Supplementary Table 4 Age standardized incidence, prevalence, DALYs, and deaths rate of gallbladder and biliary tract cancer in 1990 and 2021, and estimated annual percentage change (EAPC) from 1990 to 2021 by 204 country.

Supplementary Table 5 Frontier analysis of ASIR from 1990 to 2021.

Supplementary Table 6 Frontier analysis of ASPR from 1990 to 2021.

Supplementary Table 7 Frontier analysis of ASDR from 1990 to 2021.

Supplementary Table 8 Frontier analysis of ASMR from 1990 to 2021.

Supplementary Table 9 Decomposition analysis of incident cases.

Supplementary Table 10 Decomposition analysis of prevalent cases.

Supplementary Table 11 Decomposition analysis of DALYs cases.

Supplementary Table 12 Decomposition analysis of deaths cases.
