# Supplementary material for: Global burden and cross-country inequalities of gallbladder and biliary tract cancer in adults aged 45 years and older from 1990 to 2021: population-based study
Source: Front Oncol. 2025 Oct 9;15:1676636. doi: 10.3389/fonc.2025.1676636 (PMC12545154; doi:10.3389/fonc.2025.1676636)
Supplement: Supplementary Figure 1 — Global and SDI-stratified ASPR (Age-Standardized Prevalence Rate) trends of GBTC from 1990 to 2021. [file DataSheet1.zip › Supplementary Figure&Table/Table S4 Age standardized incidence, prevalence, DALYs, and deaths rate of of gallbladder and biliary tract cancer in 1990 and 2021, and estimated annual percentage change (EAPC) from 1990 to 2021 by 204 cou.docx]

**Table S4** Age standardized incidence, prevalence, DALYs, and deaths rate of of gallbladder and biliary tract cancer in 1990 and 2021, and estimated annual percentage change (EAPC) from 1990 to 2021 by 204 countries and territories

|  | **Age standardized rate in 2021 (per 100 000 population) (95% CI)** | | | | **EAPC in age standardized rate (%) from 1990 to 2021 (95% CI)** | | | |
| --- | --- | --- | --- | --- | --- | --- | --- | --- |
|  | **Incidence** | **Prevalence** | **DALYs** | **Deaths** | **Incidence** | **Prevalence** | **DALYs** | **Deaths** |
| Afghanistan | 6.184(2.808,10.683) | 5.917(2.598,10.298) | 157.486(67.171,274.869) | 6.666(3.098,11.398) | 1.123(1.003,1.244) | 1.143(1.021,1.265) | 1.002(0.883,1.121) | 1.142(1.019,1.265) |
| Albania | 4.559(2.891,7.032) | 4.689(2.965,7.343) | 88.842(56.004,138.617) | 4.671(2.965,7.117) | -0.044(-0.233,0.145) | 0.234(0.058,0.411) | -0.457(-0.644,-0.269) | -0.291(-0.483,-0.099) |
| Algeria | 11.354(7.347,16.731) | 11.837(7.748,17.429) | 228.52(149.185,334.013) | 11.661(7.625,16.997) | -0.161(-0.320,-0.002) | 0.086(-0.072,0.245) | -0.623(-0.747,-0.498) | -0.329(-0.493,-0.165) |
| American Samoa | 1.702(1.141,2.667) | 1.712(1.146,2.681) | 39.408(26.491,61.819) | 1.786(1.204,2.794) | -0.921(-1.338,-0.502) | -0.811(-1.220,-0.399) | -0.923(-1.337,-0.508) | -1.007(-1.425,-0.588) |
| Andorra | 10.092(6.135,15.072) | 17.878(10.821,27.107) | 123.696(76.798,182.023) | 6.274(3.949,9.223) | -0.611(-0.820,-0.402) | 0.111(-0.170,0.392) | -1.427(-1.601,-1.252) | -1.330(-1.506,-1.154) |
| Angola | 1.312(0.806,1.98) | 1.24(0.763,1.867) | 31.876(19.819,47.613) | 1.437(0.888,2.165) | 0.774(0.652,0.897) | 0.870(0.740,1.000) | 0.648(0.520,0.777) | 0.769(0.647,0.892) |
| Antigua and Barbuda | 3.982(3.453,4.545) | 4.209(3.636,4.823) | 83.427(72.712,95.261) | 4.012(3.49,4.585) | -2.648(-2.967,-2.329) | -2.509(-2.822,-2.196) | -2.919(-3.236,-2.600) | -2.746(-3.064,-2.427) |
| Argentina | 11.659(9.856,13.646) | 13.197(11.15,15.462) | 250.975(213.869,291.568) | 11.221(9.489,13.1) | -1.961(-2.105,-1.816) | -1.563(-1.692,-1.434) | -2.043(-2.166,-1.919) | -2.253(-2.410,-2.095) |
| Armenia | 5.602(4.663,6.653) | 5.815(4.828,6.914) | 124.565(102.901,147.358) | 5.732(4.746,6.775) | 2.969(2.083,3.862) | 3.116(2.216,4.023) | 2.715(1.862,3.575) | 2.883(2.009,3.766) |
| Australia | 11.596(9.412,13.764) | 32.496(26.418,38.632) | 71.288(61.502,80.418) | 3.573(3.024,4.063) | -0.264(-0.523,-0.004) | 0.805(0.380,1.231) | -2.206(-2.324,-2.087) | -2.060(-2.172,-1.949) |
| Austria | 8.259(6.701,9.866) | 12.872(10.349,15.568) | 116.661(97.611,136.668) | 5.947(4.895,7.012) | -2.189(-2.396,-1.983) | -1.080(-1.266,-0.893) | -3.094(-3.338,-2.850) | -3.120(-3.370,-2.869) |
| Azerbaijan | 2.344(1.37,3.6) | 2.314(1.352,3.551) | 55.64(32.123,85.559) | 2.463(1.431,3.784) | -0.797(-0.938,-0.656) | -0.832(-0.981,-0.682) | -1.074(-1.212,-0.935) | -0.803(-0.940,-0.666) |
| Bahamas | 5.091(4.002,6.342) | 5.334(4.189,6.685) | 117.688(92.197,148.133) | 5.229(4.137,6.501) | -1.807(-1.987,-1.627) | -1.705(-1.888,-1.522) | -2.032(-2.223,-1.841) | -1.870(-2.047,-1.693) |
| Bahrain | 3.53(2.187,5.268) | 3.941(2.421,5.917) | 62.419(38.743,94.318) | 3.313(2.076,4.899) | -0.261(-0.556,0.035) | 0.335(0.075,0.595) | -1.044(-1.293,-0.793) | -0.702(-1.028,-0.375) |
| Bangladesh | 6.463(4.025,10.355) | 6.517(4.048,10.459) | 151.498(93.341,245.984) | 6.879(4.285,11.003) | 0.284(0.113,0.455) | 0.468(0.336,0.600) | 0.056(-0.090,0.203) | 0.197(0.000,0.395) |
| Barbados | 4.639(3.5,5.912) | 4.932(3.691,6.311) | 97.401(72.801,124.845) | 4.672(3.532,5.917) | -1.078(-1.227,-0.928) | -0.917(-1.061,-0.773) | -1.284(-1.427,-1.141) | -1.204(-1.356,-1.051) |
| Belarus | 5.242(4.05,6.709) | 6.028(4.633,7.767) | 105.497(81.296,135.018) | 4.806(3.709,6.129) | 0.474(0.311,0.637) | 0.817(0.660,0.974) | -0.055(-0.262,0.153) | 0.158(-0.035,0.352) |
| Belgium | 4.584(3.656,5.602) | 7.627(6.039,9.391) | 61.782(50.847,73.224) | 3.063(2.473,3.659) | -2.000(-2.234,-1.766) | -1.045(-1.283,-0.806) | -2.664(-2.864,-2.464) | -2.797(-3.022,-2.572) |
| Belize | 3.524(2.958,4.168) | 3.665(3.072,4.346) | 84.371(70.76,99.85) | 3.663(3.075,4.321) | -2.085(-2.455,-1.714) | -1.950(-2.284,-1.615) | -2.115(-2.487,-1.743) | -2.171(-2.560,-1.780) |
| Benin | 0.144(0.068,0.229) | 0.128(0.062,0.203) | 3.084(1.534,4.899) | 0.165(0.076,0.263) | 1.508(0.896,2.123) | 1.342(0.716,1.972) | 1.030(0.406,1.659) | 1.641(1.038,2.248) |
| Bermuda | 2.64(2.028,3.44) | 3.755(2.852,4.904) | 42.518(32.877,54.919) | 2.077(1.612,2.675) | -3.172(-3.389,-2.955) | -2.171(-2.384,-1.958) | -4.083(-4.318,-3.847) | -3.969(-4.191,-3.747) |
| Bhutan | 7.505(4.675,11.487) | 7.372(4.59,11.31) | 173.217(107.724,266.811) | 8.072(5.051,12.286) | 0.378(0.333,0.423) | 0.474(0.425,0.523) | 0.085(0.032,0.137) | 0.334(0.291,0.377) |
| Bolivia (Plurinational State of) | 19.487(11.639,30.248) | 18.822(11.244,29.17) | 454.299(268.865,697.541) | 21.118(12.527,32.26) | -0.971(-1.072,-0.870) | -0.897(-1.000,-0.794) | -1.186(-1.292,-1.080) | -1.005(-1.101,-0.910) |
| Bosnia and Herzegovina | 11.078(7.656,19.127) | 11.751(8.12,20.402) | 228.477(158.249,394.455) | 11.063(7.679,18.892) | -2.019(-2.267,-1.769) | -1.747(-1.965,-1.530) | -2.398(-2.642,-2.153) | -2.214(-2.488,-1.941) |
| Botswana | 2.264(1.381,3.682) | 2.108(1.287,3.429) | 52.829(31.816,87.026) | 2.485(1.508,4.059) | 0.335(0.058,0.613) | 0.390(0.096,0.684) | 0.202(-0.077,0.482) | 0.333(0.052,0.614) |
| Brazil | 7.993(7.198,8.605) | 8.449(7.678,9.065) | 181.332(166.329,193.854) | 8.187(7.341,8.832) | -1.219(-1.371,-1.068) | -0.968(-1.130,-0.805) | -1.366(-1.516,-1.216) | -1.378(-1.518,-1.238) |
| Brunei Darussalam | 8.968(6.056,12.636) | 8.772(5.985,12.358) | 186.033(127.168,262.122) | 9.519(6.413,13.411) | -0.834(-1.016,-0.651) | -0.691(-0.844,-0.538) | -1.147(-1.315,-0.978) | -0.909(-1.109,-0.708) |
| Bulgaria | 5.878(4.706,7.334) | 7.733(6.105,9.739) | 102.69(82.646,127.196) | 4.754(3.839,5.874) | -0.197(-0.406,0.012) | 0.305(0.147,0.463) | -0.658(-0.890,-0.426) | -0.615(-0.873,-0.356) |
| Burkina Faso | 0.173(0.08,0.272) | 0.152(0.073,0.239) | 3.692(1.827,5.805) | 0.199(0.091,0.314) | 1.672(1.063,2.285) | 1.541(0.907,2.178) | 1.265(0.627,1.907) | 1.795(1.198,2.395) |
| Burundi | 2.567(1.456,4.031) | 2.403(1.359,3.787) | 62.041(35.045,98.839) | 2.838(1.622,4.464) | -1.366(-1.520,-1.212) | -1.237(-1.376,-1.098) | -1.522(-1.685,-1.359) | -1.327(-1.475,-1.178) |
| Cabo Verde | 1.255(0.454,1.997) | 1.224(0.443,1.95) | 26.364(9.371,42.186) | 1.347(0.476,2.164) | 3.787(3.088,4.491) | 3.866(3.172,4.565) | 3.488(2.766,4.216) | 3.712(3.001,4.428) |
| Cambodia | 3.622(2.114,7.801) | 3.557(2.081,7.568) | 86.776(51.492,180.016) | 3.855(2.277,8.236) | -0.146(-0.406,0.115) | -0.030(-0.293,0.234) | -0.386(-0.631,-0.142) | -0.207(-0.462,0.048) |
| Cameroon | 0.179(0.083,0.288) | 0.16(0.077,0.257) | 3.857(1.881,6.255) | 0.204(0.092,0.332) | 1.573(0.970,2.180) | 1.420(0.790,2.055) | 1.163(0.559,1.770) | 1.695(1.101,2.291) |
| Canada | 6.113(4.919,7.419) | 11.739(9.349,14.448) | 65.751(55.301,76.921) | 3.43(2.831,4.035) | -1.462(-1.587,-1.337) | -0.656(-0.847,-0.465) | -2.504(-2.601,-2.406) | -2.277(-2.366,-2.189) |
| Central African Republic | 1.189(0.68,2.089) | 1.086(0.618,1.91) | 29.868(16.74,52.533) | 1.313(0.746,2.293) | -0.200(-0.238,-0.162) | -0.181(-0.217,-0.145) | -0.260(-0.299,-0.221) | -0.174(-0.213,-0.135) |
| Chad | 0.151(0.079,0.24) | 0.135(0.072,0.213) | 3.381(1.829,5.309) | 0.173(0.088,0.27) | 2.178(1.637,2.723) | 2.030(1.476,2.587) | 1.828(1.293,2.365) | 2.291(1.751,2.834) |
| Chile | 31.861(26.653,37.63) | 41.64(34.689,49.602) | 583.379(494.86,679.73) | 27.107(22.719,31.789) | -2.333(-2.531,-2.135) | -1.559(-1.748,-1.370) | -3.103(-3.325,-2.881) | -2.961(-3.171,-2.751) |
| China | 8.581(5.767,11.217) | 12.578(8.304,16.506) | 134.889(92.879,178.318) | 6.527(4.465,8.538) | 0.457(0.355,0.559) | 1.940(1.765,2.115) | -0.867(-0.971,-0.763) | -0.749(-0.868,-0.631) |
| Colombia | 8.805(6.911,10.894) | 9.966(7.825,12.393) | 188.105(148.095,231.593) | 8.559(6.733,10.499) | -1.972(-2.216,-1.727) | -1.571(-1.845,-1.297) | -2.361(-2.617,-2.105) | -2.249(-2.468,-2.030) |
| Comoros | 2.763(1.592,4.47) | 2.624(1.513,4.252) | 66.028(37.553,108.169) | 3.034(1.739,4.928) | 0.008(-0.052,0.068) | 0.056(-0.006,0.118) | -0.130(-0.199,-0.061) | 0.020(-0.035,0.076) |
| Congo | 1.429(0.906,2.101) | 1.351(0.853,1.988) | 34.769(21.795,51.67) | 1.559(0.988,2.306) | -0.016(-0.100,0.068) | 0.103(0.016,0.190) | -0.129(-0.213,-0.045) | -0.027(-0.106,0.052) |
| Cook Islands | 1.794(1.101,2.868) | 2.122(1.294,3.382) | 35.508(21.589,56.497) | 1.662(1.019,2.635) | -1.481(-1.689,-1.272) | -0.904(-1.114,-0.694) | -1.927(-2.144,-1.710) | -1.915(-2.122,-1.707) |
| Costa Rica | 7.13(5.718,8.647) | 8.418(6.764,10.244) | 147.323(119.188,178.82) | 6.668(5.368,8.093) | -2.786(-3.119,-2.451) | -2.410(-2.754,-2.065) | -3.013(-3.353,-2.673) | -3.060(-3.382,-2.737) |
| Croatia | 0.168(0.078,0.263) | 0.15(0.072,0.234) | 3.585(1.749,5.636) | 0.191(0.087,0.3) | -0.345(-0.452,-0.238) | 0.761(0.642,0.880) | -1.455(-1.568,-1.341) | -1.318(-1.421,-1.215) |
| Cuba | 13.772(10.977,16.965) | 21.221(16.68,26.526) | 186.644(153.398,224.876) | 9.595(7.88,11.562) | -2.134(-2.292,-1.975) | -1.803(-1.962,-1.642) | -2.394(-2.562,-2.226) | -2.395(-2.553,-2.237) |
| Cyprus | 3.492(2.81,4.251) | 4.115(3.28,5.034) | 70.109(56.65,85.154) | 3.24(2.622,3.928) | -1.309(-1.452,-1.165) | 0.387(0.147,0.628) | -2.541(-2.615,-2.466) | -2.699(-2.819,-2.578) |
| Czechia | 7.319(4.75,10.67) | 11.382(7.429,16.69) | 91.429(60.721,133.128) | 5.066(3.3,7.342) | -2.054(-2.194,-1.914) | -1.172(-1.369,-0.975) | -2.815(-2.903,-2.728) | -2.756(-2.862,-2.650) |
| Côte d'Ivoire | 15.735(12.641,19.065) | 20.849(16.687,25.478) | 260.429(212.946,310.951) | 12.889(10.447,15.451) | 1.785(1.228,2.344) | 1.679(1.091,2.271) | 1.354(0.794,1.917) | 1.893(1.349,2.440) |
| Democratic People's Republic of Korea | 4.977(2.915,9.089) | 5.33(3.125,9.649) | 115.795(67.772,206.02) | 4.988(2.934,9.053) | -0.477(-0.550,-0.404) | -0.153(-0.251,-0.055) | -0.633(-0.693,-0.572) | -0.690(-0.761,-0.620) |
| Democratic Republic of the Congo | 1.075(0.618,1.775) | 1.014(0.587,1.665) | 26.226(15.068,43.47) | 1.18(0.67,1.982) | 0.339(0.170,0.507) | 0.389(0.204,0.575) | 0.271(0.106,0.436) | 0.336(0.173,0.500) |
| Denmark | 6.885(5.507,8.443) | 13.857(10.993,17.241) | 70.781(58.812,83.941) | 3.658(3.002,4.356) | -0.032(-0.246,0.183) | 1.457(1.196,1.718) | -1.763(-1.955,-1.570) | -1.519(-1.728,-1.309) |
| Djibouti | 2.753(1.557,4.663) | 2.603(1.465,4.432) | 65.244(36.263,110.558) | 3.028(1.705,5.047) | 0.173(0.120,0.226) | 0.215(0.169,0.261) | 0.077(0.010,0.144) | 0.169(0.112,0.226) |
| Dominica | 5.694(3.883,7.98) | 5.622(3.847,7.899) | 129.81(88.52,181.933) | 6.088(4.162,8.515) | -1.036(-1.131,-0.941) | -0.976(-1.075,-0.877) | -1.074(-1.165,-0.983) | -1.065(-1.156,-0.974) |
| Dominican Republic | 2.797(1.906,4.193) | 2.838(1.932,4.255) | 65.986(45.108,97.912) | 2.952(2.016,4.412) | -0.497(-0.697,-0.296) | -0.425(-0.615,-0.235) | -0.545(-0.754,-0.336) | -0.595(-0.803,-0.387) |
| Ecuador | 10.403(7.895,13.482) | 10.659(8.033,13.888) | 227.557(170.404,298.918) | 10.91(8.278,14.138) | -1.399(-1.549,-1.248) | -1.260(-1.403,-1.116) | -1.683(-1.857,-1.509) | -1.510(-1.666,-1.354) |
| Egypt | 4.873(3.271,6.838) | 4.873(3.268,6.838) | 107.217(70.796,150.257) | 5.061(3.397,7.131) | 1.739(1.403,2.077) | 1.822(1.522,2.123) | 1.365(1.047,1.684) | 1.633(1.274,1.994) |
| El Salvador | 8.803(6.15,12.73) | 9.446(6.558,13.629) | 201.492(139.178,291.717) | 8.872(6.161,12.83) | -0.725(-0.917,-0.534) | -0.472(-0.664,-0.280) | -0.991(-1.197,-0.785) | -0.934(-1.123,-0.745) |
| Equatorial Guinea | 1.333(0.755,2.146) | 1.289(0.727,2.082) | 31.262(17.384,51.196) | 1.433(0.815,2.318) | 0.575(0.476,0.674) | 0.746(0.641,0.850) | 0.289(0.189,0.389) | 0.511(0.422,0.601) |
| Eritrea | 3.467(2.01,5.522) | 3.206(1.857,5.114) | 84.028(48.218,134.72) | 3.838(2.214,6.138) | 0.306(0.250,0.363) | 0.374(0.318,0.430) | 0.177(0.127,0.227) | 0.339(0.278,0.401) |
| Estonia | 6.8(5.415,8.325) | 8.283(6.544,10.184) | 120.232(96.939,146.302) | 6(4.819,7.301) | -0.661(-0.887,-0.434) | -0.023(-0.255,0.209) | -1.515(-1.772,-1.258) | -1.170(-1.406,-0.933) |
| Eswatini | 3.233(1.856,5.23) | 3.013(1.724,4.88) | 78.982(44.611,129.154) | 3.524(2.012,5.711) | 1.333(0.934,1.733) | 1.306(0.968,1.645) | 1.355(0.907,1.805) | 1.323(0.925,1.721) |
| Ethiopia | 4.571(3.014,6.712) | 4.333(2.855,6.391) | 108.178(70.87,161.226) | 5.039(3.323,7.414) | -1.482(-1.697,-1.266) | -1.339(-1.547,-1.130) | -1.742(-1.955,-1.528) | -1.458(-1.668,-1.248) |
| Fiji | 3.652(2.133,5.377) | 3.544(2.061,5.249) | 86.544(49.812,128.164) | 3.926(2.282,5.747) | 0.468(0.324,0.613) | 0.524(0.362,0.687) | 0.386(0.214,0.559) | 0.446(0.310,0.583) |
| Finland | 9.179(7.345,11.121) | 14.443(11.493,17.685) | 127.46(105.334,150.958) | 6.469(5.227,7.71) | -1.229(-1.455,-1.003) | -0.195(-0.375,-0.014) | -2.151(-2.410,-1.891) | -2.095(-2.362,-1.826) |
| France | 5.185(4.047,6.369) | 8.759(6.828,10.899) | 64.504(52.486,77.019) | 3.405(2.718,4.091) | -2.254(-2.344,-2.165) | -1.036(-1.176,-0.896) | -3.412(-3.492,-3.332) | -3.345(-3.430,-3.260) |
| Gabon | 1.459(0.892,2.188) | 1.389(0.847,2.074) | 34.13(20.661,51.109) | 1.586(0.968,2.378) | -0.165(-0.261,-0.069) | -0.088(-0.170,-0.006) | -0.302(-0.410,-0.195) | -0.194(-0.294,-0.094) |
| Gambia | 0(0,0) | 0(0,0) | 0.003(0.001,0.004) | 0(0,0) | 0.103(-0.025,0.230) | 0.118(-0.023,0.259) | 0.020(-0.130,0.170) | 0.095(-0.023,0.212) |
| Georgia | 5.361(4.24,6.646) | 5.455(4.311,6.763) | 126.356(98.698,156.089) | 5.567(4.37,6.85) | -1.552(-2.773,-0.315) | -1.631(-2.862,-0.384) | -1.670(-2.855,-0.471) | -1.467(-2.687,-0.233) |
| Germany | 11.693(9.552,13.92) | 20.876(16.884,25.181) | 144.379(121.86,166.878) | 7.236(6,8.415) | -2.065(-2.383,-1.745) | -0.816(-1.048,-0.583) | -3.084(-3.489,-2.676) | -3.158(-3.581,-2.733) |
| Ghana | 0.181(0.085,0.285) | 0.161(0.078,0.254) | 3.809(1.912,5.967) | 0.206(0.095,0.325) | 2.532(1.872,3.195) | 2.362(1.685,3.044) | 2.054(1.386,2.726) | 2.657(2.004,3.313) |
| Greece | 6.702(5.696,7.738) | 10.485(8.787,12.33) | 95.741(84.242,107.526) | 4.768(4.125,5.367) | -0.682(-1.397,0.038) | -0.198(-0.968,0.578) | -0.943(-1.570,-0.312) | -1.023(-1.685,-0.355) |
| Greenland | 6.165(4.102,9.111) | 6.827(4.518,10.193) | 125.584(82.656,188.675) | 5.972(3.947,8.873) | -2.364(-2.582,-2.145) | -1.879(-2.122,-1.637) | -2.715(-2.909,-2.520) | -2.644(-2.846,-2.442) |
| Grenada | 4.772(3.854,5.805) | 4.841(3.909,5.898) | 109.266(88.073,133.47) | 4.961(4.008,6.048) | -1.206(-1.418,-0.994) | -1.082(-1.262,-0.901) | -1.318(-1.463,-1.173) | -1.284(-1.510,-1.058) |
| Guam | 0.966(0.694,1.352) | 1.131(0.814,1.572) | 23.097(16.707,31.965) | 0.906(0.649,1.263) | -1.317(-1.525,-1.108) | -0.897(-1.109,-0.683) | -1.116(-1.316,-0.917) | -1.619(-1.842,-1.394) |
| Guatemala | 5.554(4.595,6.653) | 5.528(4.568,6.64) | 132.235(109.111,158.904) | 5.961(4.934,7.12) | -4.359(-4.684,-4.033) | -4.135(-4.464,-3.805) | -4.388(-4.724,-4.050) | -4.464(-4.782,-4.144) |
| Guinea | 0.149(0.072,0.244) | 0.132(0.066,0.215) | 3.269(1.651,5.357) | 0.17(0.08,0.279) | 1.804(1.250,2.361) | 1.623(1.059,2.191) | 1.403(0.840,1.968) | 1.938(1.387,2.492) |
| Guinea-Bissau | 0.21(0.103,0.336) | 0.183(0.092,0.292) | 4.633(2.393,7.414) | 0.24(0.115,0.387) | 1.903(1.304,2.506) | 1.726(1.122,2.335) | 1.427(0.826,2.031) | 2.056(1.460,2.655) |
| Guyana | 3.647(2.781,4.72) | 3.595(2.729,4.678) | 91.842(69.249,119.939) | 3.883(2.966,5) | -1.599(-1.822,-1.375) | -1.478(-1.708,-1.248) | -1.569(-1.791,-1.346) | -1.646(-1.863,-1.429) |
| Haiti | 5.12(2.933,8.187) | 4.857(2.762,7.797) | 128.031(72.045,205.777) | 5.606(3.223,8.903) | -0.965(-1.014,-0.917) | -0.920(-0.999,-0.840) | -1.021(-1.073,-0.969) | -0.968(-1.015,-0.920) |
| Honduras | 11.355(7.074,16.799) | 11.126(6.924,16.515) | 275.132(170.348,411.271) | 12.21(7.604,18.035) | 1.207(1.026,1.389) | 1.204(1.042,1.365) | 1.090(0.924,1.257) | 1.170(0.981,1.359) |
| Hungary | 11.087(9.251,12.989) | 12.62(10.5,14.81) | 224.773(188.592,262.019) | 10.446(8.72,12.189) | -2.935(-3.065,-2.804) | -2.518(-2.664,-2.372) | -3.096(-3.201,-2.991) | -3.220(-3.334,-3.106) |
| Iceland | 5.865(4.569,7.266) | 11.248(8.662,14.214) | 64.637(52.194,77.809) | 3.35(2.669,4.047) | -1.004(-1.113,-0.894) | -0.093(-0.265,0.079) | -2.120(-2.210,-2.030) | -1.871(-1.970,-1.772) |
| India | 7.858(5.303,9.482) | 7.833(5.27,9.447) | 188.294(125.281,227.461) | 8.321(5.614,10.057) | 1.407(1.332,1.482) | 1.533(1.466,1.600) | 1.176(1.119,1.233) | 1.361(1.274,1.447) |
| Indonesia | 2.412(1.549,5.204) | 2.392(1.551,5.106) | 56.794(36.883,120.023) | 2.565(1.629,5.576) | -0.331(-0.373,-0.288) | -0.273(-0.327,-0.220) | -0.559(-0.595,-0.522) | -0.371(-0.408,-0.335) |
| Iran (Islamic Republic of) | 3.043(1.641,3.67) | 3.487(1.845,4.241) | 59.73(32.639,71.049) | 2.898(1.576,3.46) | 2.819(2.320,3.320) | 3.262(2.784,3.742) | 2.412(1.889,2.938) | 2.481(1.963,3.002) |
| Iraq | 3.78(2.459,5.427) | 4.032(2.623,5.801) | 81.667(52.905,118.075) | 3.767(2.458,5.386) | 0.101(-0.081,0.283) | 0.312(0.100,0.525) | -0.379(-0.502,-0.256) | -0.086(-0.244,0.072) |
| Ireland | 4.373(3.446,5.373) | 7.792(6.08,9.692) | 51.655(42.186,62.038) | 2.692(2.157,3.245) | -1.846(-2.151,-1.540) | -0.397(-0.706,-0.087) | -3.320(-3.659,-2.980) | -3.135(-3.470,-2.798) |
| Israel | 3.964(3.187,4.825) | 5.998(4.8,7.364) | 56.437(46.478,67.72) | 2.934(2.372,3.543) | -2.133(-2.309,-1.956) | -1.115(-1.310,-0.919) | -3.037(-3.196,-2.877) | -2.955(-3.125,-2.784) |
| Italy | 11.476(9.817,12.943) | 16.792(14.2,19.226) | 169.044(151.24,183.694) | 8.784(7.634,9.645) | -0.832(-1.002,-0.661) | -0.057(-0.309,0.195) | -1.717(-1.830,-1.604) | -1.482(-1.595,-1.369) |
| Jamaica | 3.744(2.746,4.97) | 3.988(2.912,5.323) | 84.43(61.005,113.231) | 3.797(2.77,5.029) | -1.126(-1.408,-0.842) | -1.019(-1.295,-0.743) | -1.240(-1.541,-0.937) | -1.207(-1.488,-0.924) |
| Japan | 22.513(18.623,25.569) | 33.993(28.144,39.218) | 286.807(252.798,307.635) | 16.528(13.937,18.065) | -1.336(-1.400,-1.273) | -0.619(-0.720,-0.518) | -2.334(-2.381,-2.287) | -1.938(-1.984,-1.892) |
| Jordan | 4.555(2.885,6.825) | 5.205(3.3,7.82) | 89.104(56.768,134.158) | 4.33(2.767,6.474) | -0.984(-1.235,-0.733) | -0.425(-0.647,-0.204) | -1.636(-1.929,-1.342) | -1.386(-1.659,-1.113) |
| Kazakhstan | 3.796(3.108,4.547) | 3.882(3.172,4.654) | 89.515(73.515,107.31) | 3.919(3.22,4.69) | -0.889(-1.090,-0.689) | -0.737(-0.897,-0.578) | -1.070(-1.302,-0.838) | -0.974(-1.201,-0.747) |
| Kenya | 3.976(2.803,5.75) | 3.751(2.649,5.438) | 93.449(65.845,136.082) | 4.38(3.081,6.333) | 0.887(0.741,1.032) | 0.874(0.722,1.026) | 0.798(0.651,0.946) | 0.914(0.767,1.061) |
| Kiribati | 2.07(0.965,3.209) | 1.92(0.9,2.978) | 50.581(23.327,79.053) | 2.283(1.05,3.567) | 0.201(0.115,0.287) | 0.214(0.135,0.293) | 0.112(0.035,0.189) | 0.217(0.128,0.306) |
| Kuwait | 4.585(3.436,5.901) | 6.353(4.706,8.251) | 74.378(56.721,94.932) | 3.701(2.787,4.712) | -0.727(-1.851,0.409) | -0.011(-1.159,1.150) | -1.558(-2.663,-0.440) | -1.318(-2.421,-0.202) |
| Kyrgyzstan | 3.638(2.847,4.598) | 3.697(2.885,4.691) | 87.855(68.335,111.448) | 3.8(2.975,4.802) | -2.356(-3.562,-1.136) | -2.243(-3.467,-1.003) | -2.468(-3.704,-1.216) | -2.426(-3.619,-1.218) |
| Lao People's Democratic Republic | 2.854(1.636,6.36) | 2.773(1.59,6.139) | 69.616(39.873,153.161) | 3.083(1.766,6.953) | -1.048(-1.215,-0.881) | -0.933(-1.101,-0.765) | -1.248(-1.406,-1.090) | -1.063(-1.228,-0.897) |
| Latvia | 4.672(3.76,5.689) | 5.205(4.197,6.367) | 95.531(77.506,116.309) | 4.446(3.592,5.407) | -0.155(-0.359,0.050) | 0.074(-0.130,0.278) | -0.528(-0.748,-0.307) | -0.315(-0.522,-0.107) |
| Lebanon | 5.524(3.35,8.022) | 6.672(4.043,9.763) | 98.716(60.633,143.135) | 5.011(3.015,7.286) | -0.288(-0.461,-0.115) | 0.392(0.229,0.556) | -1.084(-1.252,-0.915) | -0.818(-1.005,-0.631) |
| Lesotho | 3.282(1.902,5.372) | 2.975(1.721,4.876) | 80.723(46.71,131.563) | 3.599(2.105,5.851) | 2.899(2.448,3.352) | 2.764(2.348,3.183) | 2.962(2.487,3.439) | 2.886(2.435,3.339) |
| Liberia | 0.147(0.069,0.244) | 0.133(0.064,0.22) | 3.201(1.574,5.265) | 0.166(0.077,0.275) | 1.544(0.849,2.244) | 1.477(0.765,2.194) | 1.148(0.435,1.866) | 1.619(0.932,2.310) |
| Libya | 12.642(8.031,18.634) | 13.538(8.651,19.986) | 278.235(175.878,411.62) | 12.702(8.018,18.724) | 0.621(0.417,0.825) | 0.845(0.624,1.066) | 0.338(0.169,0.508) | 0.429(0.238,0.622) |
| Lithuania | 7.693(6.125,9.437) | 9.817(7.72,12.205) | 139.029(112.379,168.854) | 6.559(5.287,7.956) | -0.486(-0.956,-0.013) | -0.127(-0.618,0.366) | -0.882(-1.306,-0.457) | -0.752(-1.201,-0.301) |
| Luxembourg | 5.372(4.442,6.395) | 9.048(7.403,10.929) | 68.095(57.613,78.381) | 3.535(2.964,4.087) | -1.504(-1.656,-1.353) | -0.278(-0.433,-0.124) | -2.676(-2.854,-2.498) | -2.566(-2.752,-2.379) |
| Madagascar | 2.22(1.299,3.432) | 2.096(1.227,3.24) | 54.252(31.699,83.719) | 2.434(1.426,3.749) | -0.167(-0.257,-0.078) | -0.155(-0.255,-0.055) | -0.248(-0.337,-0.159) | -0.158(-0.246,-0.071) |
| Malawi | 0.906(0.528,1.43) | 0.843(0.489,1.337) | 21.498(12.407,34.235) | 1.001(0.585,1.575) | -0.029(-0.159,0.102) | 0.047(-0.051,0.145) | -0.169(-0.302,-0.035) | -0.006(-0.140,0.128) |
| Malaysia | 3.984(2.506,5.483) | 4.32(2.689,5.954) | 87.028(54.098,119.715) | 3.951(2.501,5.431) | -0.676(-0.849,-0.503) | -0.349(-0.522,-0.176) | -1.034(-1.221,-0.847) | -0.911(-1.085,-0.737) |
| Maldives | 2.099(1.268,3.066) | 2.296(1.392,3.384) | 40.777(24.941,60.4) | 2.052(1.233,2.999) | -2.165(-2.311,-2.019) | -1.686(-1.823,-1.549) | -2.980(-3.153,-2.806) | -2.532(-2.687,-2.377) |
| Mali | 0.811(0.518,1.267) | 0.766(0.489,1.201) | 19.402(12.303,30.702) | 0.892(0.571,1.404) | 0.658(0.398,0.918) | 0.709(0.446,0.971) | 0.557(0.294,0.820) | 0.660(0.403,0.917) |
| Malta | 3.816(3.02,4.725) | 6.223(4.893,7.772) | 52.038(42.151,62.914) | 2.619(2.095,3.176) | -1.671(-1.781,-1.561) | -0.616(-0.727,-0.505) | -2.558(-2.675,-2.442) | -2.537(-2.648,-2.426) |
| Marshall Islands | 2.148(1.316,3.219) | 2.066(1.253,3.13) | 53.114(31.739,81.799) | 2.324(1.425,3.488) | -0.637(-0.723,-0.551) | -0.542(-0.623,-0.461) | -0.637(-0.736,-0.538) | -0.677(-0.766,-0.588) |
| Mauritania | 0.147(0.071,0.244) | 0.135(0.067,0.224) | 3.118(1.549,5.202) | 0.165(0.077,0.274) | 1.050(0.374,1.730) | 1.006(0.315,1.703) | 0.596(-0.095,1.293) | 1.108(0.447,1.773) |
| Mauritius | 2.805(2.417,3.189) | 3.061(2.636,3.481) | 60.663(52.692,68.575) | 2.77(2.39,3.146) | -3.971(-5.558,-2.359) | -3.695(-5.297,-2.065) | -4.179(-5.741,-2.592) | -4.148(-5.719,-2.551) |
| Mexico | 7.628(6.663,8.578) | 8.039(7.006,9.062) | 174.416(151.806,197.064) | 7.805(6.832,8.761) | -2.871(-3.066,-2.676) | -2.634(-2.842,-2.425) | -2.963(-3.166,-2.759) | -3.045(-3.228,-2.862) |
| Micronesia (Federated States of) | 2.207(1.387,3.275) | 2.122(1.329,3.165) | 53.605(33.24,80.547) | 2.375(1.485,3.545) | -0.885(-0.938,-0.831) | -0.778(-0.834,-0.721) | -0.958(-1.014,-0.902) | -0.942(-0.994,-0.890) |
| Monaco | 3.295(2.151,4.87) | 5.642(3.648,8.423) | 41.847(27.895,60.944) | 2.111(1.412,3.069) | -0.033(-0.111,0.044) | 0.557(0.460,0.654) | -0.694(-0.758,-0.630) | -0.599(-0.664,-0.534) |
| Mongolia | 9.457(6.342,14.594) | 9.23(6.193,14.237) | 233.687(157.491,358.512) | 10.094(6.807,15.582) | -0.904(-1.114,-0.693) | -0.876(-1.122,-0.629) | -1.228(-1.463,-0.993) | -0.888(-1.083,-0.692) |
| Montenegro | 5.897(4.059,8.044) | 6.452(4.433,8.879) | 110.728(76.747,151.97) | 5.672(3.907,7.753) | 0.256(0.119,0.394) | 0.256(0.136,0.376) | -0.100(-0.279,0.080) | 0.213(0.051,0.375) |
| Morocco | 2.368(1.363,3.453) | 2.389(1.377,3.507) | 54.256(30.912,79.35) | 2.478(1.42,3.596) | 0.344(0.262,0.427) | 0.466(0.376,0.555) | 0.153(0.077,0.228) | 0.251(0.172,0.329) |
| Mozambique | 3.145(1.708,5.12) | 2.883(1.554,4.702) | 75.807(41.143,124.132) | 3.482(1.919,5.7) | 1.133(0.970,1.295) | 1.096(0.937,1.254) | 1.093(0.928,1.258) | 1.139(0.976,1.302) |
| Myanmar | 2.406(1.373,5.189) | 2.399(1.373,5.119) | 57.583(33.119,121.87) | 2.57(1.466,5.578) | -1.162(-1.302,-1.022) | -0.994(-1.147,-0.840) | -1.401(-1.529,-1.273) | -1.203(-1.337,-1.069) |
| Namibia | 1.888(1.169,2.867) | 1.803(1.11,2.728) | 44.411(26.962,67.791) | 2.047(1.265,3.124) | 0.002(-0.240,0.245) | 0.073(-0.137,0.282) | -0.138(-0.407,0.132) | -0.025(-0.270,0.221) |
| Nauru | 2.931(1.627,4.513) | 2.83(1.56,4.379) | 72.038(39.325,112.754) | 3.133(1.74,4.846) | -0.642(-0.746,-0.537) | -0.581(-0.668,-0.495) | -0.704(-0.832,-0.575) | -0.674(-0.783,-0.564) |
| Nepal | 7.543(4.804,11.418) | 7.334(4.674,11.094) | 178.785(114.255,269.872) | 8.153(5.212,12.228) | 0.948(0.778,1.119) | 1.030(0.864,1.196) | 0.741(0.568,0.913) | 0.916(0.752,1.079) |
| Netherlands | 8.751(7.097,10.579) | 16.988(13.638,20.959) | 99.128(83.151,116.032) | 5.018(4.151,5.9) | -0.578(-0.777,-0.379) | 0.768(0.536,1.001) | -1.897(-2.119,-1.674) | -1.894(-2.139,-1.649) |
| New Zealand | 12.531(9.945,15.453) | 28.619(22.184,36.07) | 116.238(98.006,135.584) | 5.746(4.75,6.753) | 1.285(0.774,1.799) | 2.640(2.141,3.142) | -0.301(-0.798,0.199) | -0.201(-0.702,0.302) |
| Nicaragua | 7.412(5.033,11.577) | 7.832(5.345,12.232) | 172.008(117.609,268.368) | 7.6(5.168,11.812) | -1.191(-1.307,-1.074) | -1.013(-1.116,-0.911) | -1.366(-1.471,-1.262) | -1.343(-1.472,-1.214) |
| Niger | 0.133(0.066,0.22) | 0.118(0.06,0.195) | 2.912(1.464,4.854) | 0.152(0.073,0.255) | 1.525(0.890,2.164) | 1.394(0.751,2.041) | 1.121(0.481,1.765) | 1.634(1.006,2.266) |
| Nigeria | 0.146(0.097,0.213) | 0.139(0.09,0.202) | 3.479(2.214,5.07) | 0.161(0.108,0.234) | 0.833(0.578,1.088) | 0.912(0.640,1.185) | 0.768(0.512,1.025) | 0.810(0.564,1.057) |
| Niue | 2.122(1.355,3.044) | 2.149(1.365,3.099) | 47.431(30.186,68.615) | 2.209(1.417,3.166) | -0.707(-0.779,-0.635) | -0.538(-0.610,-0.466) | -0.879(-0.957,-0.801) | -0.831(-0.903,-0.759) |
| North Macedonia | 8.825(6.041,12.057) | 8.789(6.034,12.096) | 175.642(121.421,241.182) | 9.116(6.276,12.401) | -0.620(-0.862,-0.379) | -0.502(-0.709,-0.294) | -1.078(-1.292,-0.864) | -0.742(-1.002,-0.482) |
| Northern Mariana Islands | 1.435(0.972,2.557) | 1.555(1.056,2.785) | 31.104(21.176,55.498) | 1.428(0.969,2.525) | 0.029(-0.578,0.640) | 0.154(-0.475,0.786) | -0.051(-0.635,0.536) | -0.058(-0.654,0.542) |
| Norway | 6.954(5.806,8.092) | 15.125(12.561,17.83) | 66.758(59.92,72.517) | 3.385(2.97,3.713) | -0.012(-0.264,0.240) | 1.650(1.369,1.932) | -1.915(-2.203,-1.626) | -1.785(-2.067,-1.502) |
| Oman | 2.669(1.694,4.022) | 3.053(1.94,4.597) | 51.191(32.921,77.216) | 2.45(1.56,3.67) | -0.329(-0.485,-0.173) | 0.104(-0.027,0.236) | -0.976(-1.170,-0.781) | -0.704(-0.889,-0.518) |
| Pakistan | 11.262(7.687,15.963) | 10.734(7.326,15.243) | 272.932(186.224,392.047) | 12.288(8.436,17.543) | 0.151(-0.053,0.355) | 0.161(-0.032,0.356) | 0.027(-0.192,0.247) | 0.158(-0.048,0.365) |
| Palau | 1.462(0.89,2.298) | 1.512(0.913,2.38) | 33.903(20.213,54.051) | 1.505(0.915,2.382) | -0.320(-0.387,-0.252) | -0.162(-0.236,-0.087) | -0.476(-0.542,-0.410) | -0.411(-0.473,-0.349) |
| Palestine | 4.742(2.949,6.565) | 4.916(3.065,6.793) | 97.603(61.013,135.43) | 4.81(2.99,6.669) | -0.498(-0.693,-0.303) | -0.253(-0.393,-0.111) | -0.804(-1.006,-0.603) | -0.687(-0.914,-0.460) |
| Panama | 4.309(3.292,5.33) | 4.819(3.665,5.964) | 92.088(70.279,113.975) | 4.239(3.227,5.224) | -2.414(-2.603,-2.224) | -2.140(-2.349,-1.931) | -2.587(-2.772,-2.402) | -2.611(-2.783,-2.439) |
| Papua New Guinea | 1.53(0.952,2.318) | 1.49(0.925,2.261) | 37.682(23.353,57.619) | 1.658(1.033,2.518) | -0.924(-0.993,-0.855) | -0.875(-0.951,-0.799) | -0.987(-1.058,-0.917) | -0.921(-0.986,-0.856) |
| Paraguay | 6.619(4.278,9.705) | 6.85(4.441,10.072) | 147.555(95.786,215.406) | 6.897(4.467,10.082) | -0.010(-0.125,0.104) | 0.098(-0.018,0.214) | -0.171(-0.263,-0.079) | -0.104(-0.216,0.009) |
| Peru | 14.595(9.467,21.701) | 16.178(10.548,24.097) | 315.363(205.246,470.401) | 14.384(9.388,21.399) | -0.593(-0.903,-0.283) | -0.184(-0.512,0.145) | -1.002(-1.299,-0.704) | -0.912(-1.201,-0.622) |
| Philippines | 1.737(1.339,3.021) | 1.761(1.359,3.021) | 42.296(32.513,71.875) | 1.823(1.404,3.194) | 0.379(0.346,0.412) | 0.442(0.410,0.474) | 0.340(0.307,0.372) | 0.313(0.274,0.352) |
| Poland | 10.007(8.918,10.967) | 9.886(8.841,10.824) | 225.987(203.27,246.701) | 10.82(9.624,11.842) | -2.044(-2.183,-1.905) | -1.803(-1.945,-1.661) | -2.142(-2.245,-2.039) | -2.165(-2.300,-2.030) |
| Portugal | 7.965(6.416,9.704) | 14.218(11.333,17.553) | 99.543(83.271,117.313) | 4.947(4.089,5.87) | -0.719(-0.856,-0.582) | 0.940(0.789,1.092) | -2.110(-2.283,-1.936) | -2.187(-2.357,-2.017) |
| Puerto Rico | 2.617(2.004,3.301) | 3.426(2.607,4.378) | 49.424(37.961,62.381) | 2.244(1.726,2.82) | -2.593(-2.783,-2.404) | -1.848(-2.022,-1.674) | -3.062(-3.267,-2.856) | -3.166(-3.367,-2.965) |
| Qatar | 6.011(3.699,9.311) | 7.636(4.647,12.108) | 94.794(57.829,149.697) | 5.024(3.095,7.739) | -0.530(-1.075,0.018) | 0.489(0.021,0.959) | -1.391(-1.941,-0.837) | -1.365(-1.971,-0.755) |
| Republic of Korea | 29.181(17.728,40.748) | 43.397(26.44,60.529) | 385.261(239.168,528.764) | 21.563(13.094,29.49) | -0.769(-0.844,-0.693) | 0.840(0.711,0.969) | -2.646(-2.749,-2.543) | -2.142(-2.254,-2.030) |
| Republic of Moldova | 3.133(2.675,3.645) | 3.37(2.883,3.926) | 70.624(60.663,82.12) | 3.083(2.64,3.579) | -1.512(-1.897,-1.126) | -1.271(-1.684,-0.855) | -1.654(-2.007,-1.300) | -1.674(-2.037,-1.310) |
| Romania | 5.57(4.576,6.681) | 6.068(4.963,7.283) | 122.004(99.727,146.401) | 5.468(4.483,6.538) | -1.093(-1.379,-0.807) | -0.763(-1.067,-0.458) | -1.430(-1.711,-1.149) | -1.333(-1.608,-1.057) |
| Russian Federation | 7.501(6.759,8.185) | 12.025(10.841,13.149) | 109.185(99.204,119.183) | 5.083(4.591,5.55) | -0.151(-0.531,0.230) | 1.021(0.556,1.488) | -1.406(-1.793,-1.018) | -1.182(-1.553,-0.810) |
| Rwanda | 3.222(1.936,4.928) | 3.034(1.82,4.633) | 76.204(45.43,116.557) | 3.554(2.136,5.42) | -1.325(-1.564,-1.085) | -1.111(-1.341,-0.880) | -1.601(-1.856,-1.345) | -1.289(-1.521,-1.056) |
| Saint Kitts and Nevis | 3.523(2.806,4.269) | 3.516(2.782,4.29) | 77.469(60.764,94.808) | 3.705(2.951,4.485) | -2.381(-2.552,-2.210) | -2.179(-2.347,-2.012) | -2.665(-2.832,-2.498) | -2.511(-2.683,-2.339) |
| Saint Lucia | 2.813(2.228,3.498) | 2.926(2.3,3.65) | 63.215(49.478,79.067) | 2.923(2.3,3.629) | -2.993(-3.340,-2.644) | -2.677(-2.999,-2.353) | -3.049(-3.391,-2.706) | -3.171(-3.531,-2.809) |
| Saint Vincent and the Grenadines | 2.984(2.509,3.523) | 3.027(2.539,3.592) | 69.222(58.168,81.95) | 3.149(2.658,3.699) | -2.453(-2.713,-2.193) | -2.336(-2.593,-2.078) | -2.519(-2.787,-2.251) | -2.508(-2.764,-2.251) |
| Samoa | 2.112(1.369,3.03) | 2.141(1.381,3.08) | 49.63(31.686,72.031) | 2.228(1.436,3.22) | -0.576(-0.631,-0.521) | -0.400(-0.458,-0.343) | -0.626(-0.680,-0.572) | -0.670(-0.722,-0.617) |
| San Marino | 7.202(4.156,11.476) | 12.786(7.174,20.682) | 89.495(50.689,140.763) | 4.536(2.633,7.056) | -1.349(-1.691,-1.005) | -0.798(-1.175,-0.420) | -1.764(-2.068,-1.458) | -1.818(-2.147,-1.488) |
| Sao Tome and Principe | 4.954(3.009,7.36) | 4.807(2.915,7.143) | 115.728(69.968,173.691) | 5.323(3.258,7.994) | 0.705(0.618,0.792) | 0.775(0.698,0.853) | 0.566(0.467,0.666) | 0.664(0.570,0.759) |
| Saudi Arabia | 6.009(4.054,9.115) | 6.873(4.602,10.436) | 123.089(82.962,186.316) | 5.667(3.844,8.62) | 0.346(0.157,0.535) | 0.933(0.795,1.072) | -0.100(-0.324,0.125) | -0.125(-0.357,0.107) |
| Senegal | 0.156(0.077,0.26) | 0.14(0.071,0.233) | 3.366(1.703,5.625) | 0.178(0.084,0.297) | 1.828(1.164,2.496) | 1.707(1.029,2.389) | 1.419(0.736,2.106) | 1.935(1.280,2.595) |
| Serbia | 7.79(5.149,10.908) | 8.165(5.404,11.415) | 165.179(109.5,231.908) | 7.884(5.224,11.073) | -1.177(-1.317,-1.036) | -0.670(-0.780,-0.560) | -1.394(-1.565,-1.223) | -1.552(-1.713,-1.390) |
| Seychelles | 3.103(2.184,4.633) | 3.224(2.274,4.808) | 66.732(47.197,99.221) | 3.171(2.232,4.708) | -2.196(-2.569,-1.821) | -1.992(-2.366,-1.616) | -2.501(-2.880,-2.121) | -2.353(-2.725,-1.980) |
| Sierra Leone | 0.148(0.07,0.237) | 0.131(0.064,0.21) | 3.206(1.606,5.157) | 0.168(0.079,0.271) | 1.983(1.375,2.594) | 1.859(1.233,2.489) | 1.638(1.019,2.261) | 2.072(1.472,2.675) |
| Singapore | 4.8(3.834,5.841) | 7.08(5.625,8.707) | 67.715(55.783,80.284) | 3.621(2.935,4.317) | -1.284(-1.427,-1.141) | -0.021(-0.164,0.122) | -2.659(-2.835,-2.484) | -2.338(-2.498,-2.177) |
| Slovakia | 20.29(13.595,29.406) | 32.974(21.896,48.104) | 278.807(189.409,398.106) | 13.432(9.082,19.304) | -0.499(-0.609,-0.388) | 0.384(0.231,0.538) | -1.490(-1.568,-1.413) | -1.310(-1.383,-1.237) |
| Slovenia | 12.727(10.002,15.918) | 21.189(16.469,27.037) | 164.569(131.813,202.761) | 8.365(6.683,10.305) | -0.809(-0.973,-0.645) | 0.676(0.453,0.899) | -2.435(-2.575,-2.295) | -2.147(-2.305,-1.988) |
| Solomon Islands | 1.969(1.197,3.019) | 1.892(1.144,2.903) | 49.221(29.364,75.866) | 2.129(1.287,3.257) | -0.454(-0.570,-0.339) | -0.360(-0.492,-0.228) | -0.473(-0.607,-0.339) | -0.491(-0.594,-0.388) |
| Somalia | 2.745(1.493,4.886) | 2.539(1.382,4.516) | 68.633(36.949,122.421) | 3.019(1.63,5.389) | 0.228(0.185,0.271) | 0.254(0.212,0.296) | 0.163(0.118,0.208) | 0.254(0.209,0.298) |
| South Africa | 2.812(1.82,3.414) | 2.741(1.779,3.327) | 65.682(42.925,79.682) | 3.002(1.934,3.637) | 0.931(0.755,1.108) | 0.971(0.818,1.125) | 0.890(0.702,1.079) | 0.845(0.655,1.035) |
| South Sudan | 2.692(1.582,4.245) | 2.538(1.486,4.022) | 66.169(38.295,105.309) | 2.944(1.726,4.63) | 0.322(0.176,0.468) | 0.375(0.224,0.526) | 0.305(0.149,0.461) | 0.310(0.172,0.448) |
| Spain | 10.295(8.189,12.693) | 22.914(17.931,28.627) | 94.172(77.782,110.83) | 4.81(3.891,5.713) | -0.986(-1.140,-0.833) | 0.377(0.195,0.558) | -2.557(-2.737,-2.376) | -2.499(-2.677,-2.320) |
| Sri Lanka | 2.838(1.568,5.425) | 3.266(1.787,6.212) | 57.469(31.904,105.525) | 2.684(1.512,4.959) | -5.030(-5.819,-4.234) | -4.513(-5.319,-3.700) | -5.507(-6.312,-4.695) | -5.442(-6.222,-4.656) |
| Sudan | 3.062(1.923,4.786) | 3.057(1.907,4.793) | 72.693(45.125,113.915) | 3.249(2.061,5.047) | 0.370(0.269,0.471) | 0.475(0.373,0.578) | 0.156(0.063,0.249) | 0.325(0.225,0.426) |
| Suriname | 3.09(2.024,4.519) | 3.12(2.048,4.559) | 74.931(49.193,108.918) | 3.276(2.152,4.802) | -1.174(-1.319,-1.028) | -1.085(-1.229,-0.941) | -1.247(-1.404,-1.091) | -1.221(-1.361,-1.080) |
| Sweden | 9.33(7.63,11.048) | 10.58(8.703,12.569) | 173.858(144.099,205.221) | 8.975(7.326,10.622) | -2.569(-3.049,-2.087) | -2.843(-3.399,-2.284) | -2.401(-2.791,-2.009) | -2.206(-2.619,-1.791) |
| Switzerland | 9.135(6.896,11.624) | 21.992(16.431,28.406) | 70.323(56.204,85.568) | 3.682(2.887,4.509) | 0.038(-0.356,0.434) | 0.956(0.436,1.478) | -1.384(-1.632,-1.136) | -1.137(-1.432,-0.841) |
| Syrian Arab Republic | 0.307(0.174,0.437) | 0.332(0.188,0.476) | 6.359(3.52,9.131) | 0.303(0.169,0.43) | 0.881(0.596,1.167) | 1.202(0.900,1.505) | 0.426(0.144,0.709) | 0.595(0.323,0.867) |
| Taiwan (Province of China) | 10.424(8.482,12.553) | 16.138(12.956,19.657) | 160.33(133.46,188.779) | 7.606(6.263,9) | -0.862(-1.606,-0.112) | 0.046(-0.776,0.875) | -2.024(-2.700,-1.344) | -1.669(-2.350,-0.983) |
| Tajikistan | 0.389(0.243,0.571) | 0.372(0.234,0.544) | 9.543(6.044,14.003) | 0.419(0.263,0.617) | -1.782(-1.965,-1.598) | -1.841(-2.000,-1.682) | -1.783(-1.948,-1.618) | -1.784(-1.980,-1.588) |
| Thailand | 24.64(12.415,36.35) | 30.027(14.937,44.828) | 507.4(253.525,755.019) | 22.497(11.488,33.199) | 0.122(0.037,0.207) | 0.802(0.716,0.888) | -0.360(-0.438,-0.283) | -0.392(-0.479,-0.304) |
| Timor-Leste | 2.208(1.27,4.816) | 2.157(1.241,4.651) | 53.459(30.916,114.331) | 2.378(1.364,5.176) | -0.580(-0.850,-0.308) | -0.484(-0.753,-0.214) | -0.728(-1.004,-0.451) | -0.631(-0.897,-0.363) |
| Togo | 0.175(0.08,0.291) | 0.156(0.073,0.259) | 3.748(1.785,6.229) | 0.199(0.088,0.333) | 2.139(1.496,2.786) | 1.975(1.312,2.642) | 1.699(1.054,2.349) | 2.269(1.634,2.908) |
| Tokelau | 1.901(1.2,2.841) | 1.933(1.215,2.913) | 43.725(27.386,65.776) | 1.986(1.259,2.963) | -1.046(-1.102,-0.990) | -0.857(-0.914,-0.799) | -1.237(-1.289,-1.186) | -1.169(-1.224,-1.114) |
| Tonga | 1.62(1.013,2.469) | 1.65(1.029,2.517) | 37.256(23.19,56.626) | 1.697(1.063,2.58) | -0.491(-0.621,-0.360) | -0.423(-0.542,-0.303) | -0.662(-0.785,-0.538) | -0.549(-0.684,-0.414) |
| Trinidad and Tobago | 3.465(2.595,4.481) | 3.643(2.71,4.74) | 80.299(59.723,104.661) | 3.553(2.674,4.579) | -2.759(-3.106,-2.412) | -2.505(-2.853,-2.156) | -2.920(-3.276,-2.562) | -2.904(-3.245,-2.563) |
| Tunisia | 7.977(4.881,12.165) | 9.077(5.571,13.806) | 158.576(97.765,243.942) | 7.604(4.75,11.613) | -0.096(-0.127,-0.064) | 0.333(0.292,0.375) | -0.475(-0.517,-0.432) | -0.443(-0.488,-0.399) |
| Turkey | 5.131(3.54,7.388) | 5.926(4.064,8.478) | 99.911(68.81,142.655) | 4.85(3.368,6.942) | -5.250(-6.523,-3.961) | -5.155(-6.433,-3.860) | -5.252(-6.503,-3.984) | -5.295(-6.564,-4.008) |
| Turkmenistan | 2.644(1.998,3.474) | 2.652(2,3.494) | 66.609(50.429,88.25) | 2.772(2.116,3.635) | -1.023(-1.095,-0.950) | -0.887(-0.956,-0.817) | -1.169(-1.243,-1.095) | -1.077(-1.148,-1.006) |
| Tuvalu | 2.018(1.32,2.905) | 1.975(1.292,2.855) | 48.305(31.462,69.19) | 2.163(1.407,3.09) | -1.135(-1.359,-0.912) | -0.578(-0.804,-0.351) | -1.857(-2.044,-1.668) | -1.579(-1.793,-1.366) |
| Uganda | 2.702(1.677,4.048) | 2.545(1.568,3.816) | 63.845(38.748,97.033) | 2.973(1.839,4.471) | -0.188(-0.407,0.031) | -0.079(-0.285,0.128) | -0.319(-0.561,-0.076) | -0.180(-0.397,0.038) |
| Ukraine | 3.943(2.818,5.286) | 4.653(3.308,6.274) | 85.003(60.396,114.827) | 3.562(2.562,4.767) | -0.001(-0.247,0.245) | 0.382(0.123,0.641) | -0.292(-0.526,-0.057) | -0.297(-0.528,-0.065) |
| United Arab Emirates | 14.178(9.338,21.058) | 14.552(9.592,21.547) | 274.066(178.189,409.705) | 14.561(9.491,21.647) | 1.095(0.496,1.697) | 1.111(0.555,1.669) | 0.247(-0.282,0.778) | 1.068(0.440,1.700) |
| United Kingdom | 8.814(7.909,9.429) | 20.901(18.931,22.362) | 72.59(67.003,76.177) | 3.678(3.3,3.896) | 1.584(1.196,1.973) | 2.805(2.441,3.171) | -0.107(-0.530,0.317) | 0.011(-0.419,0.443) |
| United Republic of Tanzania | 2.796(1.689,4.208) | 2.648(1.596,3.999) | 66.611(40.093,101.417) | 3.066(1.872,4.623) | 0.007(-0.034,0.048) | 0.081(0.024,0.139) | -0.077(-0.120,-0.034) | 0.003(-0.034,0.041) |
| United States of America | 7.569(6.801,8.094) | 18.163(16.471,19.375) | 65.583(60.569,69.021) | 3.089(2.769,3.281) | -0.504(-0.570,-0.437) | 0.208(0.120,0.296) | -1.302(-1.456,-1.148) | -1.413(-1.561,-1.265) |
| United States Virgin Islands | 3.121(2.002,4.716) | 3.292(2.102,4.995) | 67.336(43.106,101.337) | 3.144(2.03,4.697) | -3.087(-3.350,-2.824) | -2.997(-3.276,-2.717) | -3.246(-3.514,-2.977) | -3.184(-3.435,-2.932) |
| Uruguay | 14.909(12.293,17.755) | 17.314(14.287,20.674) | 291.583(242.616,345.649) | 13.968(11.529,16.635) | -1.242(-1.369,-1.115) | -0.884(-1.021,-0.746) | -1.605(-1.736,-1.475) | -1.516(-1.636,-1.397) |
| Uzbekistan | 1.293(0.987,1.66) | 1.251(0.955,1.609) | 31.857(24.474,41.184) | 1.365(1.052,1.757) | 1.372(0.750,1.998) | 1.356(0.695,2.021) | 1.418(0.804,2.037) | 1.319(0.707,1.935) |
| Vanuatu | 1.775(1.124,2.642) | 1.696(1.067,2.533) | 43.499(27.241,65.591) | 1.931(1.228,2.876) | -0.644(-0.732,-0.557) | -0.589(-0.687,-0.490) | -0.670(-0.774,-0.566) | -0.662(-0.743,-0.581) |
| Venezuela (Bolivarian Republic of) | 5.635(4.101,7.434) | 5.885(4.255,7.812) | 126.932(91.539,169.462) | 5.799(4.243,7.632) | -3.035(-3.402,-2.666) | -2.868(-3.217,-2.517) | -3.303(-3.681,-2.923) | -3.149(-3.529,-2.768) |
| Viet Nam | 4.51(2.801,6.945) | 4.897(3.037,7.551) | 94.637(58.638,147.852) | 4.442(2.773,6.864) | 0.742(0.688,0.797) | 1.133(1.056,1.210) | 0.362(0.315,0.409) | 0.421(0.380,0.462) |
| Yemen | 3.488(2.078,5.584) | 3.398(2.019,5.465) | 83.904(49.609,133.884) | 3.757(2.243,5.963) | 0.618(0.483,0.752) | 0.695(0.555,0.835) | 0.476(0.343,0.608) | 0.592(0.458,0.726) |
| Zambia | 3.18(1.92,4.688) | 2.983(1.797,4.406) | 76.557(45.246,112.879) | 3.486(2.079,5.126) | 0.196(0.107,0.285) | 0.276(0.181,0.371) | 0.047(-0.045,0.140) | 0.210(0.123,0.296) |
| Zimbabwe | 4.079(2.638,6.039) | 3.802(2.447,5.629) | 100.92(64.564,149.901) | 4.455(2.891,6.611) | 0.939(0.575,1.305) | 0.939(0.600,1.280) | 1.149(0.737,1.563) | 0.924(0.569,1.281) |

EAPC = estimated annual percentage change; SDI = socio-demographic index; 95% CI = 95% confidence interval.
