# Supplementary material for: Global burden and cross-country inequalities of gallbladder and biliary tract cancer in adults aged 45 years and older from 1990 to 2021: population-based study
Source: Front Oncol. 2025 Oct 9;15:1676636. doi: 10.3389/fonc.2025.1676636 (PMC12545154; doi:10.3389/fonc.2025.1676636)
Supplement: Supplementary Figure 1 — Global and SDI-stratified ASPR (Age-Standardized Prevalence Rate) trends of GBTC from 1990 to 2021. [file DataSheet1.zip › Supplementary Figure&Table/Table S2 Age standardized DALYs rate (ASDR) of gallbladder and biliary tract cancer in 1990 and 2021, and estimated annual percentage change (EAPC) from 1990 to 2021 at the global and regional level.docx]

**Table S2** Age standardized DALYs rate (ASDR) of gallbladder and biliary tract cancer in 1990 and 2021, and estimated annual percentage change (EAPC) from 1990 to 2021 at the global and regional level.

|  | **1990** | | **2021** | | **1990-2021** |
| --- | --- | --- | --- | --- | --- |
|  | **DALYs cases,**  **(95% CI)** | **ASDRs**  **per 100 000**  **(95% CI)** | **DALYs cases,**  **(95% CI)** | **ASDRs**  **per 100 000**  **(95% CI)** | **EAPC, %,**  **(95% CI)** |
| Global | 2134408.031(1868674.147,2392046.99) | 200.403(175.453,224.222) | 3499957.219(2839313.77,4028701.804) | 147.646(119.796,169.849) | -1.080(-1.123,-1.037) |
| SDI |  |  |  |  |  |
| High | 802946.951(733195.689,849074.639) | 265.584(242.132,281.082) | 885950.275(754308.797,979096.76) | 150.264(128.898,165.437) | -1.959(-2.016,-1.903) |
| High-middle | 577311.751(490409.103,628243.442) | 212.804(180.766,231.689) | 812150.457(621792.782,948744.904) | 147.393(112.848,172.155) | -1.339(-1.409,-1.269) |
| Middle | 469313.38(397190.596,595577.275) | 167.401(141.96,212.838) | 1036821.446(827417.475,1298512.394) | 137.873(110.155,172.947) | -0.741(-0.802,-0.680) |
| Low-middle | 224075.786(185375.21,319490.038) | 133.106(110.185,189.759) | 615396.531(471855.592,765199.312) | 155.392(119.529,193.656) | 0.569(0.529,0.608) |
| Low | 57628.869(44061.7,80361.879) | 91.477(70.246,127.798) | 146738.35(100041.096,186605.858) | 107.803(73.432,136.612) | 0.590(0.539,0.642) |
| Regions |  |  |  |  |  |
| Andean Latin America | 23282.086(17575.828,28750.219) | 422.561(319.651,521.416) | 50233.91(36517.118,68785.771) | 311.948(226.889,426.774) | -1.207(-1.370,-1.044) |
| Australasia | 8374.927(7622.331,9138.324) | 130.944(118.974,143.006) | 11484.717(10015.364,12777.581) | 78.518(68.988,87.126) | -1.849(-2.007,-1.691) |
| Caribbean | 9232.6(7802.809,10710.495) | 131.124(110.819,152.088) | 11482.452(9474.189,13794.499) | 77.353(63.836,92.909) | -1.889(-2.012,-1.766) |
| Central Asia | 11985.736(10489.808,13955.477) | 92.587(80.885,107.955) | 15772.128(13807.097,18027.538) | 69.133(60.612,78.91) | -1.477(-1.929,-1.023) |
| Central Europe | 144364.334(134583.699,151940.562) | 348.722(324.5,367.468) | 117987.967(106806.635,129301.69) | 194.047(175.8,212.669) | -2.052(-2.104,-1.999) |
| Central Latin America | 79950.673(76287.827,83363.781) | 357.245(340.059,373.038) | 118406.682(104287.978,133506.627) | 170.889(150.541,192.595) | -2.732(-2.929,-2.534) |
| Central Sub-Saharan Africa | 1674.156(1082.236,2593.329) | 26.921(17.555,41.494) | 4237.305(2724.786,6276.257) | 28.213(18.112,42.134) | 0.288(0.168,0.409) |
| East Asia | 411408.954(311557.411,530078.66) | 175.507(132.567,225.643) | 834718.914(586888.606,1100913.123) | 135.094(94.799,177.698) | -0.894(-1.001,-0.787) |
| Eastern Europe | 95960.074(88504.41,104744.309) | 122.609(112.904,133.854) | 99977.113(90507.187,109661.289) | 103.236(93.439,113.34) | -1.085(-1.371,-0.797) |
| Eastern Sub-Saharan Africa | 18968.343(12586.931,26346.596) | 92.364(61.98,127.531) | 35667.152(24692.518,48681.477) | 79.894(55.405,108.309) | -0.614(-0.703,-0.525) |
| High-income Asia Pacific | 333078.485(298921.472,361881.004) | 602.956(539.477,655.462) | 417146.544(345137.229,475260.835) | 305.561(257.554,348.114) | -2.340(-2.381,-2.300) |
| High-income North America | 97928.311(91456.627,102201.11) | 102.711(96.24,107.093) | 117437.393(107419.207,124150.111) | 65.652(60.399,69.258) | -1.441(-1.572,-1.310) |
| North Africa and Middle East | 52399.914(40288.365,69344.687) | 115.361(88.973,153.948) | 124647.815(89553.368,154911.117) | 101.398(73.186,126.254) | -0.241(-0.347,-0.134) |
| Oceania | 439.924(250.48,618.708) | 54.394(31.707,75.44) | 917.84(603.851,1270.186) | 44.027(29.249,60.341) | -0.701(-0.736,-0.667) |
| South Asia | 237619.968(187163.618,340238.898) | 147.868(116.761,212.433) | 782560.47(539137.563,939268.861) | 191.385(132.254,229.856) | 0.863(0.813,0.913) |
| Southeast Asia | 99226.958(71021.001,129702.191) | 142.369(102.572,186.551) | 253996.592(180376.828,336820.866) | 140.442(99.635,186.189) | -0.215(-0.282,-0.148) |
| Southern Latin America | 86799.029(78613.1,94992.386) | 684.946(619.185,750.414) | 83538.898(74005.654,93196.279) | 353.114(313.072,393.664) | -2.219(-2.320,-2.119) |
| Southern Sub-Saharan Africa | 4098.093(2964.382,5583.359) | 56.38(40.517,76.792) | 10995.174(7526.7,13294.318) | 69.447(47.29,83.892) | 0.893(0.716,1.071) |
| Tropical Latin America | 64122.143(59867.525,67717.416) | 259.905(241.259,275.161) | 128335.945(118181.961,137067.089) | 180.558(165.867,193.029) | -1.343(-1.491,-1.195) |
| Western Europe | 352591.383(324730.394,372710.713) | 221.394(204.274,233.879) | 278214.091(246523.385,301094.767) | 108.71(97.851,116.929) | -2.375(-2.534,-2.215) |
| Western Sub-Saharan Africa | 901.941(693.176,1255.35) | 3.797(2.94,5.35) | 2198.118(1444.739,2854.047) | 4.319(2.835,5.535) | 0.988(0.620,1.358) |

ASDR = age standardized deaths rate; EAPC = estimated annual percentage change; SDI = socio-demographic index; 95% CI = 95% confidence interval.
