# Supplementary figures and images for: Global burden and cross-country inequalities of gallbladder and biliary tract cancer in adults aged 45 years and older from 1990 to 2021: population-based study
Source: Front Oncol. 2025 Oct 9;15:1676636. doi: 10.3389/fonc.2025.1676636 (PMC12545154; doi:10.3389/fonc.2025.1676636)

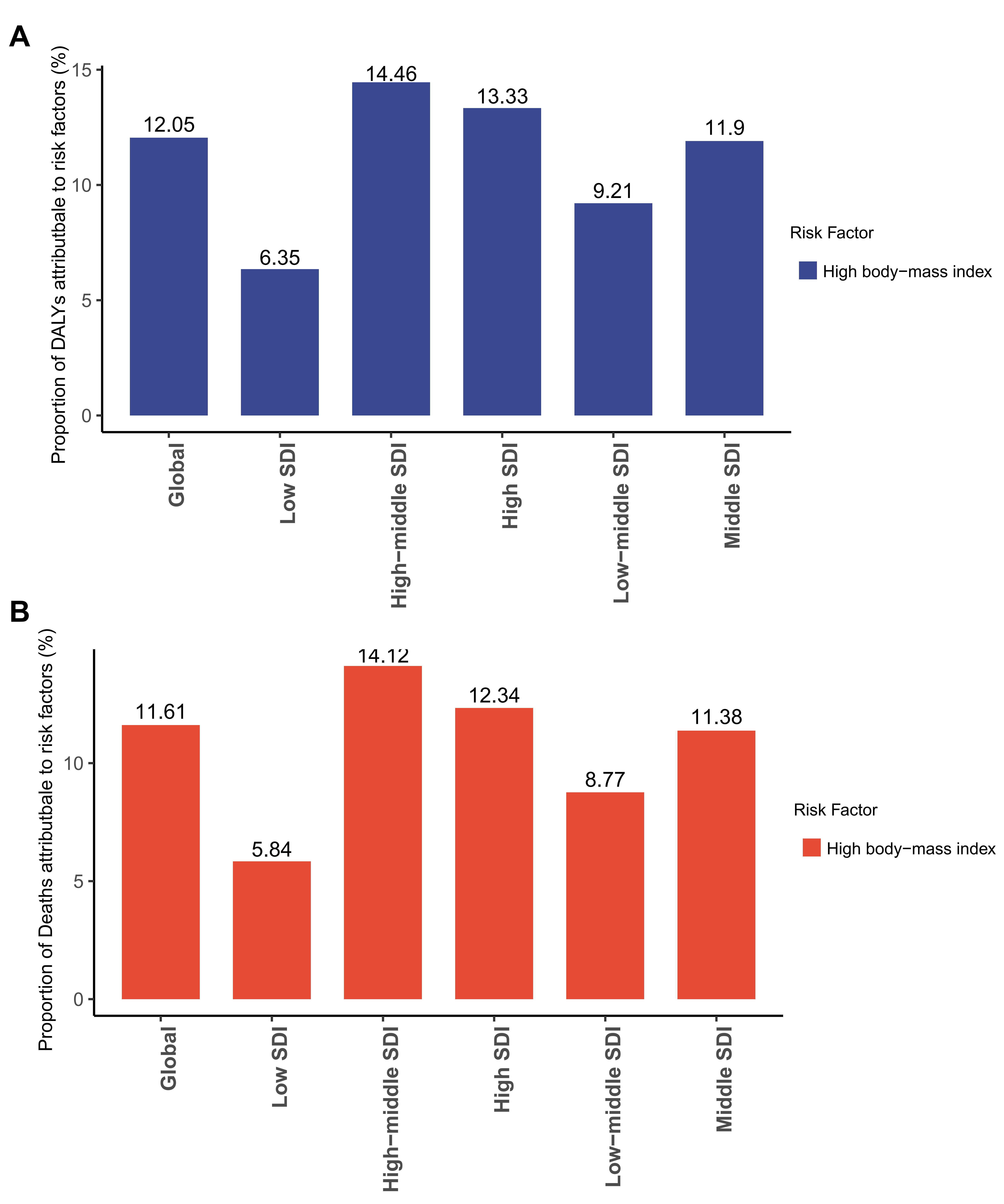

Supplement: Supplementary Figure 1 — Global and SDI-stratified ASPR (Age-Standardized Prevalence Rate) trends of GBTC from 1990 to 2021. [file DataSheet1.zip › Supplementary Figure&Table/Figure S18 Percentage of (A) DALYs and (B) deaths due to high body-mass index for the SDI regions in 2021_00.jpg]

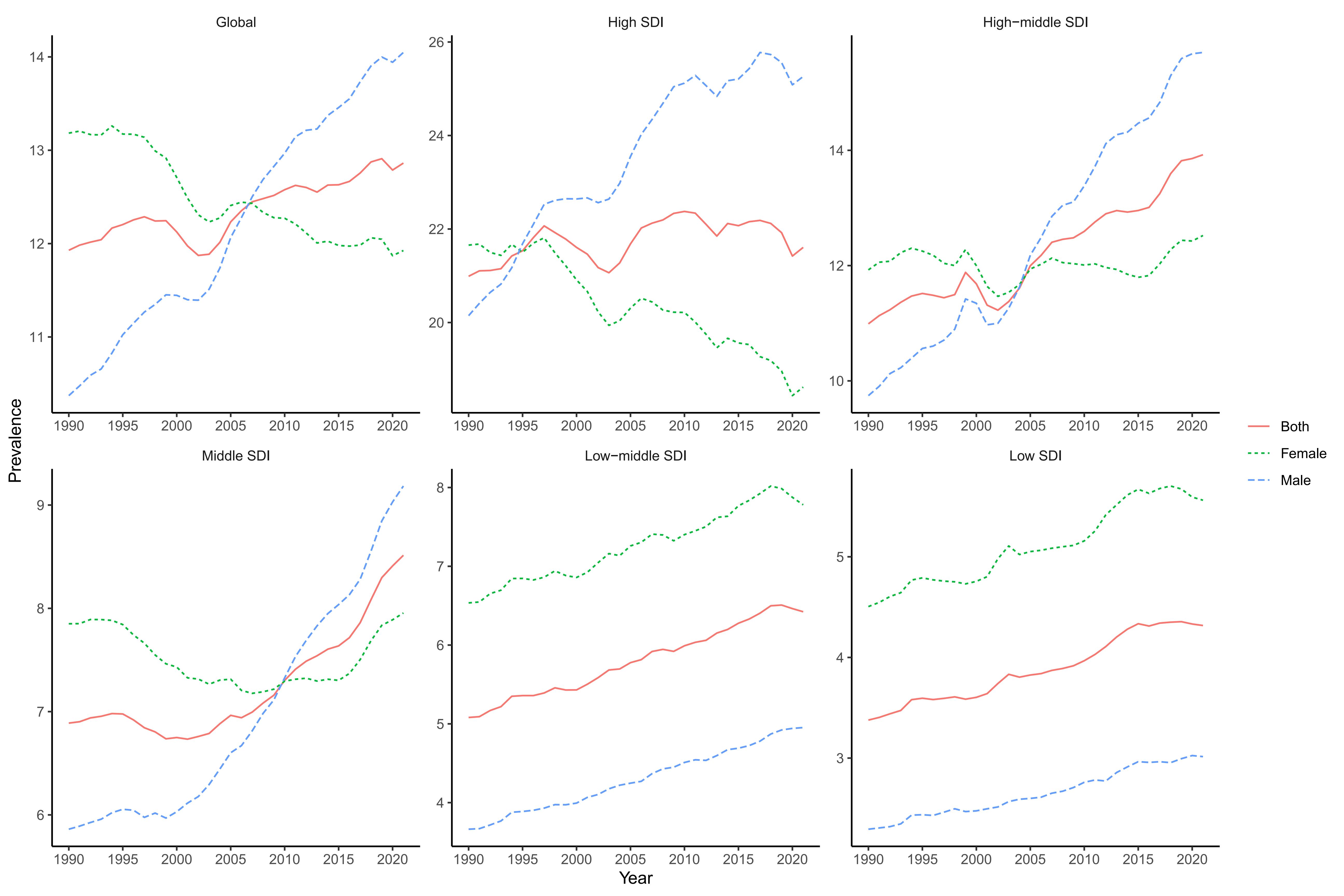

Supplement: Supplementary Figure 1 — Global and SDI-stratified ASPR (Age-Standardized Prevalence Rate) trends of GBTC from 1990 to 2021. [file DataSheet1.zip › Supplementary Figure&Table/Figure S1 Global and SDI-stratified ASPR trends of GBTC from 1990 to 2021_00.jpg]
